# Effectiveness of different exercise interventions on balance and cognitive functions in stroke patients: A network meta-analysis

**DOI:** 10.1186/s13102-025-01267-3

**Published:** 2025-08-27

**Authors:** Minghui Du, Longwei Chen, Liang Xia, Yunan Li, Enyi Ma, Zhiwei Hu, Xu Gao

**Affiliations:** 1 Sports Center, Xi’an Jiaotong University, Xi’an Shaanxi, 710049 People’s Republic of China; 2 Rehabilitation Science Institute, Shaanxi Provincial Rehabilitation Hospital, Xi’an Shaanxi, 710065 People’s Republic of China; 3 School of Physical Education, Northeast Normal University, 5268 Renmin Street, Changchun Jilin, 130024 People’s Republic of China

**Keywords:** Stroke, Exercise therapy, Balance function, Cognitive function

## Abstract

**Background:**

Exercise interventions are proven to improve functional outcomes in stroke patients, yet the optimal and safest exercise modalities remain uncertain. This network meta-analysis (NMA) aims to systematically compare the effects of various exercise interventions on balance and cognitive functions, providing robust evidence to guide clinical decision-making.

**Methods:**

Web of Science, PubMed, Embase, and Cochrane Library were searched up to September 2024. Randomized controlled trials (RCTs) evaluating exercise interventions for balance and cognitive improvements in stroke patients were included. Quality assessment and data extraction were performed, followed by Bayesian NMA using Stata 15.0 and R 4.41.

**Results:**

This study ultimately included 40 RCTs with 2,302 patients. Six commonly employed exercise interventions in clinical treatment were covered, including aerobic exercise (AE), core stability exercise (CSE), physical/mental exercise (PME), resistance training (RT), high-intensity interval training (HIIT), and mixed-component exercise (Mixed). According to the surface under the cumulative ranking curve (SUCRA), Mixed was the most effective intervention for improving Berg balance scale scores (SUCRA = 82.89%). AE was the most effective intervention for improving patients’ performance on the timed up and go test (SUCRA = 88.46%). PME exhibited superior effectiveness in improving Montreal cognitive assessment scores (SUCRA = 86.43%).

**Conclusions:**

Mixed and AE noticeably improves balance function in stroke patients, while PME and AE notably enhance cognitive function. The efficacy of other forms of exercise requires further validation. For patients whose primary objective is to improve balance, we recommend prioritizing Mixed. In cases of markedly impaired physical function, a single type of exercise should be selected. For patients aiming to enhance cognitive function, we recommend the selection of PME as the preferred option.

**Trial registration:**

Registration date: 23 September 2024.

PROSPERO registration number: CRD42024593741.

**Supplementary Information:**

The online version contains supplementary material available at 10.1186/s13102-025-01267-3.

## Background

Stroke, or acute cerebrovascular disease, is a notable contributor to neurological disabilities. Moreover, it is one of the primary causes of death and disability globally [1, 2]. As reported by the Global Burden of Disease Study, stroke prevalence and disability rates have been on the rise globally in recent years, largely due to population aging and the accumulation of risk factors [3, 4]. A study forecasts that by 2030, the global prevalence of ischemic stroke will reach 89.32 per 100,000 people. Moreover, the mortality will notably increase in low- and middle-income countries [5]. Stroke causes severe impairment of daily life functions, such as perception, sensation, language, cognition, and motor abilities, and also results in a heavy financial burden on families and society, including medical fees, nursing care costs, and productivity losses [6]. Therefore, it is essential to examine effective rehabilitation treatment methods that assist patients in their recovery.

Existing studies have demonstrated that exercise interventions can notably improve functional impairments in stroke patients, including enhancing cardiovascular endurance, balance and cognitive functions, and quality of life [7–11]. Furthermore, exercise interventions can reduce the risk of stroke recurrence and the incidence of cardiovascular events, thereby noticeably improving the survival rate and life expectancy of patients [12, 13]. These studies provide strong evidence for the application of exercise interventions in stroke rehabilitation. However, exercise interventions encompass a variety of distinct interventions, such as aerobic exercise (AE), resistance training (RT), physical/mental exercise (PME), high-intensity interval training (HIIT), and core stability exercise (CSE). Each of these exercise interventions has distinct characteristics and serves a different role in stroke treatment. For example, HIIT is marked by acute high-intensity stimuli, which are more conducive to the enhancement of cardiovascular function [11]. However, this type of training imposes greater limitations on patients’physical capabilities and has a narrower applicable population compared to moderate-intensity AE. CSE demonstrates apparent advantages in enhancing trunk functionality, increasing abdominal muscle strength, and improving balance capabilities [14]. Furthermore, when patients experience severe muscle weakness, this intervention can be conducted on the bed with the assistance of a therapist [15]. Nevertheless, compared to PME, CSE offers fewer psychological benefits and exhibits lower adherence [16]. PME can not only enhance proprioception and improve dynamic balance abilities but also facilitate increased social interaction among patients [17], thereby addressing both their psychological state and social needs [18]. Therefore, although previous studies have thoroughly analyzed the efficacy of exercise interventions, there is a lack of comparison among different types of exercise interventions. This gap in knowledge poses challenges for therapists when selecting appropriate exercise modalities while formulating exercise prescriptions.

The existing research comparing different modes of exercise primarily examines the cardiopulmonary function, motor abilities, and quality of life in stroke patients. It has been found that there are noticeable differences in the therapeutic effects of various forms of exercise on these functional aspects. For instance, Wang et al*.* [19] have employed an NMA to compare the effects of different exercise modalities in stroke patients. Their results demonstrate that HIIT exhibits the best therapeutic effects in enhancing patients’ VO_2 peak_. Moreover, RT demonstrates the most optimal effect in improving both systolic and diastolic blood pressure in patients. This further highlights that different exercise prescriptions operate through distinct mechanisms and serve different roles. Comparing the effectiveness of various exercise modalities can help develop more scientifically informed and tailored clinical exercise prescriptions.

As essential components of neurological function, cognitive and balance functions represent critical issues to focus on during rehabilitation. According to the studies by Rogge et al*.* (2018) [20] and Wittenberg et al*.* (2017) [21], balance function and cognitive function share similar neuroplasticity mechanisms. Targeted training can induce the reorganization of brain structure and function, thus improving balance function and cognitive function. This further provides a theoretical basis for integrated intervention strategies. Many studies have currently demonstrated the positive effects of exercise interventions on the recovery of balance and cognitive function in stroke patients. For example, Moreno-Segura et al*.* (2022) [22] indicate that weight transfer training, gait training, and CSE evidently improve the balance abilities of stroke patients [22]. Physical/mental exercise (PME), such as Tai Chi and Baduanjin, notably improves cognitive functions like attention, short-term memory, executive function, and visuospatial skills in both acute and chronic stroke patients [23]. However, the absence of direct and indirect comparisons between different exercise modalities restricts the scientific selection of exercise interventions in clinical treatment. To further compare the therapeutic effects of different exercise methods and provide theoretical support for stroke rehabilitation, a meta-analysis is warranted. Traditional paired meta-analysis is limited to evaluating the effects of a single intervention against a control group and cannot systematically compare the effectiveness of multiple interventions. In contrast, NMA, as a novel statistical method, can integrate both direct and indirect evidence to simultaneously evaluate and rank the effectiveness of multiple interventions, as well as quantify the differences in effects [24]. This study aims to carry out an NMA based on randomized controlled trials (RCTs) to systematically compare the effects of various exercise interventions on balance and cognitive function in stroke patients. By integrating direct and indirect evidence, it seeks to inform clinical decision-making and optimize personalized rehabilitation strategies.

## Materials and methods

### Registration information

This study was reported in accordance with the Preferred Reporting Items for Systematic Reviews and Meta-Analyses (PRISMA) guidelines (Appendix 1) [25]. The PRISMA extension statement was used to enhance the transparency and completeness of the reporting [26]. The study protocol had been registered in the International Prospective Register of Systematic Reviews (PROSPERO) (registration number: CRD42024593741).

### Inclusion and exclusion criteria

The inclusion criteria were strictly based on the PICOS (population/condition, intervention, comparison, outcomes, and study design) principles [27]. Studies must simultaneously meet the following requirements: (i) adult stroke patients with stable vital signs and no contraindications to exercise, whether ischemic or hemorrhagic stroke, and regardless of the stroke recovery phase (acute, subacute, or chronic); (ii) the intervention group that involved one or a combination of the following methods: AE, CSE, PME, RT, HIIT, and the classification of the above exercise types was determined based on the definitions provided in Table 1; (iii) the control group that only received CT (including pharmacological treatment, lifestyle interventions, physical therapy, and unstructured small-scale exercise) or any of the above interventions; (iv) studies that reported at least one of the following outcome measures: Berg balance scale (BBS), timed up and go (TUG) test, Montreal cognitive assessment (MoCA) scale, Wisconsin card sorting test (WCST), trail making test (TMT), mini-mental state examination (MMSE); (v) RCT. The following articles were excluded: (i) studies that did not specify the type of exercise intervention; (ii) conference papers, reviews, and non-RCTs (such as case reports, observational studies, cross-sectional studies, and studies without a control group); (iii) non-English literature and non-peer-reviewed publications; (iv) studies with a participant dropout rate exceeding 20%; (v) studies for which full text was inaccessible; (vi) studies with incomplete outcome data where authors had not responded after three attempts to contact.

**Table 1:** Classification Criteria for Different Types of Exercise Interventions

Type	Definition
AE	A form of exercise primarily designed to improve cardiorespiratory fitness and enhance physical capacity, with minimal focus on mental or cognitive aspects, typically including activities such as walking, cycling, and jogging in land-based settings [120]
CSE	A form of exercise focused on strengthening the core muscles, including the abdominal, lumbar, pelvic, and gluteal regions, to enhance stability and control [121]
PME	A form of exercise that integrates physical activity with inner focus, emphasizing attention to breathing, muscle sensations, and non-judgmental self-awareness [122]
RT	A form of exercise aimed at improving skeletal muscle strength, power, endurance, and size [120]
HIIT	A training method that alternates between short bursts of high-intensity exercise and periods of low-intensity recovery or rest [123]
Mixed	Mixed-component exercise interventions containing any two or more of the above exercise measures

*AE* aerobic exercise, *CSE* core stability exercise, *PME* physical/mental exercise, *RT* resistance training, *HIIT* high-intensity interval training, *Mixed* mixed-component exercise

### Search strategy

Independent searches were conducted by two investigators across Web of Science, PubMed, Embase, and Cochrane Library, covering the period from the inception of the databases to September 2024. Boolean logic operators were employed to link the subject terms with free-text terms, including “stroke”, “apoplexy”, “apoplectic stroke”, “cerebrovascular accident”, “brain accident”, “cerebrovascular injury”, “exercise”, “fitness training”, “physical activity”, “resistance exercise”, “aerobic exercise”, “high-intensity interval training”, “random”, “randomized controlled trial”, and “RCT”. The specific retrieval strategy can be found in Appendix 2. Additionally, the reference lists of pertinent meta-analyses and reviews were examined systematically to reduce the likelihood of missing any studies that met the inclusion criteria.

### Study selection process

Studies were selected independently by two investigators. They independently employed Endnote X9 software for the screening process. Initially, duplicates were removed. Subsequently, documents such as conference papers, abstracts, and letters were excluded. Systematic reviews and reviews were then organized separately. Finally, the titles, abstracts, and full texts were read in sequence to determine the studies to be included. If any important information was missing from a study, the corresponding authors would be contacted via email or other means to ensure the completeness of the data.

### Data extraction, quality evaluation and GRADE

Two investigators (MHD and LX) independently reviewed all articles and extracted data. The extracted content included basic information of each study (first author and country), participant characteristics (disease type, age, and sample size), intervention characteristics (interventions, intensity, and duration), and outcome measures (TUG, BBS, and MoCA). In the event of discrepancies during the data extraction, a third investigator (YNL) would join the discussion and assist in making the decision. Two investigators (MHD and LX) applied the second version of the risk of bias tool (ROB2) to evaluate the included articles in the following domains: (i) bias arising from the randomization process; (ii) bias due to deviations from the intended interventions; (iii) bias from missing outcome data; (iv) bias in outcome measurement; (v) selective reporting bias in results [28]. The risk of bias (ROB) was classified into three categories: low ROB, high ROB, and unclear. Furthermore, we leveraged the Grading of Recommendations Assessment, Development and Evaluation (GRADE) to appraise the quality of evidence in our NMA. This evaluation was implemented across five dimensions: limitations, inconsistency, indirectness, imprecision, and publication bias. Ultimately, the quality of evidence was categorized into four levels: high, moderate, low, and very low [29]. The evaluation process was conducted independently by two investigators. In the event of any disputes arising during the assessment, a third investigator (YNL) would be consulted to reach a consensus.

### Data synthesis

The mean difference (MD) was employed as the effect size, accompanied by a corresponding 95% credible interval (CrI). According to the methods outlined in Sect. 16.1.3.2 of the Cochrane Handbook version 5.0.2, the mean change and standard deviation before and after treatment were calculated. The extracted data were then analyzed using R version 4.4.1 and Stata version 15.0 software. Given the heterogeneity among the trials, a Bayesian hierarchical random-effects model was first used to analyze the multiple comparisons of different exercise interventions in stroke treatment [30, 31]. All calculations and graphics were performed utilizing R 4.4.1 software and Stata 15.1 software. Based on the theory of likelihood function and certain prior assumptions, Bayesian inference was conducted using R version 4.4.1 software. The Markov Chain Monte Carlo simulation was employed. A total of 500,000 iterations were set, with an annealing phase comprising 20,000 iterations, to investigate the posterior distribution of the analyzed nodes [32–34]. The overall consistency of the results was assessed by comparing the difference in the values of deviance information criterion (DIC) between the consistency model and the inconsistency model. A DIC difference of less than 5 indicated favorable consistency. The node-splitting technique was applied to evaluate the local inconsistency in results with closed loops. The connections between various treatment methods were illustrated using a network plot. Simultaneously, a corrected funnel plot was employed to assess the risk of potential publication bias [35, 36]. In addition, the exercise interventions studied were ranked utilizing the surface under the cumulative ranking curve (SUCRA). The SUCRA values ranged from 0 to 1. A higher SUCRA value indicated a higher ranking of an exercise intervention in improving specific indicators in stroke patients [37, 38]. Ultimately, a league table was generated to display the results of pairwise comparisons between each of the interventions.

## Results

### Retrieval results

**Fig. 1:**
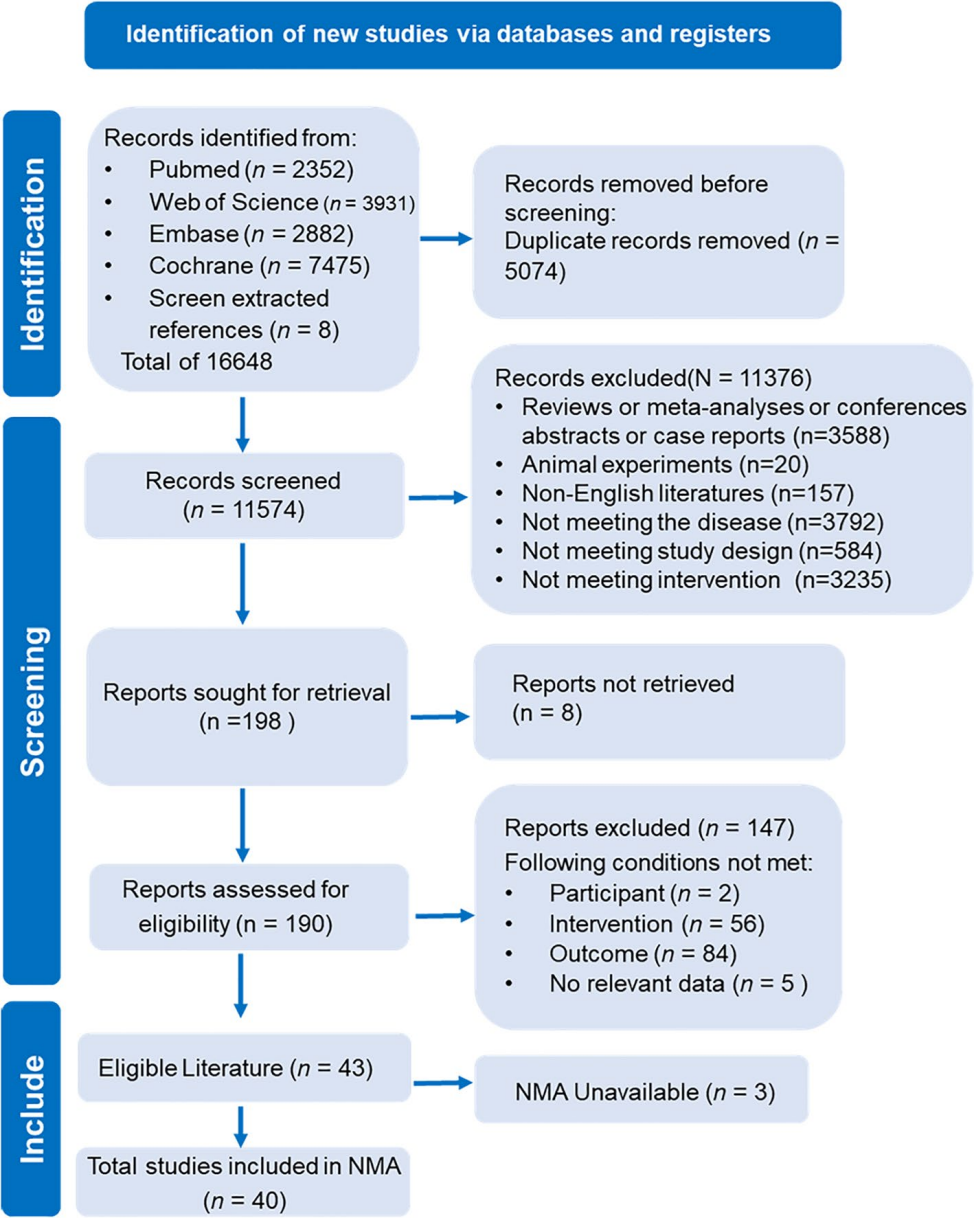
Literature screening flowchart

Through a comprehensive search of the four databases and an examination of the reference lists from other studies within the same research field, 16,648 articles were identified. After conducting a duplicate check and removing redundant entries, 11,574 unique articles remained. By reviewing titles and abstracts, we excluded non-English publications, studies with incompatible research designs, animal experiments, as well as those whose subjects or intervention methods did not meet our criteria. This process resulted in 198 remaining articles. A full-text review was executed on these 198 articles. Nevertheless, eight articles could not be accessed in full text. The remaining 190 articles underwent thorough reading to eliminate those that did not meet standards regarding study subjects, interventions, and outcome measures, as well as those from which full texts could not be obtained or lacked relevant data for extraction. Ultimately, this process yielded 43 qualifying articles. Among them, three studies had fewer than five references related to their outcome measures and were thus deemed unsuitable for NMA. In total, 40 articels were included (Fig. 1).

### Characteristics of the included studies

Ultimately, 40 RCTs [14, 39–77] were included, with 2,302 patients. In the 40 studies, 2 focused on acute stroke patients [66, 67], 1 included acute or subacute stroke patients[38], 6 targeted subacute stroke patients [14, 42, 43, 53, 54, 70], 1 involved subacute or chronic stroke patients [52], and 12 examined chronic stroke patients [41, 46, 48–51, 55, 56, 64, 67–69]. Additionally, 18 studies did not specify the recovery stage of the stroke patients [39, 40, 44, 45, 47, 57–63, 65, 71–74, 76]. Only one of the 40 RCTs specifically distinguished between ischemic and hemorrhagic strokes [72]. 10 studies focused on AE [39, 43, 46, 51, 53, 65, 67, 71, 72, 75], 12 studies examined CSE [14, 42, 44, 47, 48, 54, 56, 63, 64, 69, 70, 76], 3 studies investigated HIIT [43, 59, 65], 7 studies addressed mixed-component exercise (Mixed) [40, 41, 50, 60, 66, 71, 74], 5 studies explored PME [49, 57, 61, 62, 68], and 8 studies concentrated on RT [45, 52, 55, 58, 68, 70, 73, 77]. 27 studies [39, 41–44, 46, 50–52, 54–58, 61, 63–66, 69–73, 75–77] employed the BBS score as the outcome measure, 21 studies [14, 39, 40, 45, 47–49, 51–53, 55–58, 60, 64, 67, 68, 70, 75, 77] employed the TUG indicator, and 5 studies [59, 60, 62, 71, 74]used the MoCA indicator. The main characteristics of the 40 included articles are listed in Table 2.

**Table 2:** Basic information of the included studies

ID	Study	Country	Type	Patient	Treatment			N	Age	Gender(F/M)	Outcomes
1	Kamps 2005 [39]	German	RCT	Stroke with resulting hemiparesis	AE	MOTOmed movement therapySystem(bicycle ergometer)	2times/week, at least 10 min/time, 16 weeks	16	63.1 ± 8.1	5/11	BBS TUG
					CT	Conventional therapy	16 weeks	15	65.8 ± 10.7	4/11	
2	Langhammer 2009 [40]	Norway	RCT	Stroke	AE + RT	Endurance exercise and Strength exercises	40-60 min/day, 2-3times/week, 3 months	35	76 ± 12.7	NA	TUG
					CT	Conventional therapy	3 months	40	72 ± 13.6	NA	
3	Quaney 2009 [41]	America	RCT	Chronic stroke	AE + RT	stationary bicycle with progressive resistance	40%−70%Hrmax, 45 min/day, 3times/day, 8 weeks	19	64.1 ± 12.3	NA	BBS
					CT	Conventional therapy	8 weeks	19	58.96 ± 14.68	NA	
4	Yoo 2010 [42]	Korea	RCT	Subacute Stroke	CSE	Core strengthening program	30 min/day, 3times/weeks, 4 weeks	28	59.61 ± 18.16	15/13	BBS
					CT	Conventional therapy	4 weeks	31	61.77 ± 12.58	14/17	
5	Lau 2011 [43]	China	RCT	Subacute Stroke	HIIT	Short interval walking trials with stepwise increases in treadmill speed	30 s walking and 2 min interval, 7–8 repetitions, 30 min/time, 5times/week, 2 weeks	15	69.5 ± 11.1	5/10	BBS
					AE	Gait training on the treadmill at a steady speed	30 min/time, 5times/week, 2 weeks	15	72.1 ± 9.2	4/11	
6	Saeys 2012 [44]	Belgium	RCT	Stroke	CSE	Trunk muscle strength training	30 min/day, 4times/weeks, 8 weeks	18	61.94 ± 13.83	9/9	BBS
					CT	Conventional therapy	8 weeks	15	61.07 ± 9.01	7/8	
7	Lee 2013 [45]	Korea	RCT	Stroke	RT	Isokinetic eccentric resistance exercises	60 min/day, 3times/week, 6 weeks	10	53.40 ± 9.71	3/7	TUG
					CT	Conventional therapy	6 weeks	10	53.86 ± 10.56	4/6	
8	Jin 2013 [46]	China	RCT	chronic stroke	AE	Aerobic cycling training	50% to 70% HR reserve, 5 times/weeks, 40 min/day, 12 weeks	65	57.6 ± 6.6	19/46	BBS
					CT	Conventional therapy	12 weeks	63	56.3 ± 6.5	18/45	
9	Chung 2013 [47]	Korea	RCT	Stroke	CSE	The core stabilization exercise	30 min/day, 3times/weeks, 4 weeks	8	44.37 ± 9.90	3/5	TUG
					CT	Conventional therapy	4 weeks	8	48.38 ± 9.72	1/7	
10	Jung 2014 [48]	Korea	RCT	Chronic hemiparetic stroke	CSE	Weight-shift training	30 min/day, 5times/weeks, 4 weeks	9	51.9 ± 10.3	2/7	TUG
					CT	Conventional therapy	4 weeks	8	57.9 ± 8.5	2/6	
11	Kim 2015a [49]	Korea	RCT	Chronic stroke	PME	Tai Chi(10 Tai Chi movements)	3 to 4 movements per week, 40mim/day, 2 days/week, 6 weeks	11	53.45 ± 11.54	4/7	TUG
					CT	Conventional therapy	6 weeks	11	55.18 ± 10.20	5/6	
12	Moore 2015 [50]	England	RCT	Chronic stroke	AE + RT	Mixed exercise intervention	40%−50HRmax, 45-60 min/day, 3 days/weeks, 19 weeks	20	68 ± 8	2/18	BBS
					CT	Conventional therapy	19 weeks	20	70 ± 11	4/16	
13	Kim 2015b [51]	Korea	RCT	Chronic stroke	AE	Cycling exercise	5times/weeks, 30 min/day, 6 weeks	16	65.2 ± 6.4	4/12	BBS TUG
					CT	Conventional therapy	6 weeks	16	61.7 ± 6.1	3/13	
14	Şen 2015 [52]	Turkey	RCT	Subacute/chronic stroke	RT	Bilateral isokinetic strengthening training	5 days/weeks, 3 week	25	51.3 ± 12	8/17	BBS TUG
					CT	Conventional therapy	3 weeks	25	55.4 ± 10.5	9/16	
15	Sandberg 2016 [53]	Sweden	RCT	Subacute stroke	AE	Aerobic exercise	50%−80HRmax, 60 min/day, 2times/week, 12 weeks	29	71.3 ± 7.0	15/14	TUG
					CT	Conventional therapy	12 weeks	27	70.4 ± 8.1	13/14	
16	Cabanas-Valdés 2016 [54]	Spain	RCT	Subacute stroke	CSE	Core stability exercises	15 min/day, 5times/week, 5 weeks	40	74.92 ± 10.70	21/18	BBS
					CT	Conventional therapy	5 weeks	40	75.69 ± 9.40	19/21	
17	Fernandez-Gonzalo 2016 [55]	Sweden	RCT	Chronic stroke	RT	Flywheel resistance exercise training	12 weeks, 2 times/week; 4 sets of 7 maximal closed-chain knee extensions; < 2 min of contractile activity per session	14	61.2 ± 9.8	3/11	BBS TUG
					CT	Conventional therapy	12 weeks	15	65.7 ± 12.7	4/11	
18	An 2017 [56]	Korea	RCT	Chronic stroke	CSE	four supine exercises and seven sitting exercises	30 min/day, 3times/weeks, 4 weeks	15	59.73 ± 8.94	8/7	BBS TUG
					CT	Conventional therapy	4 weeks	14	57.07 ± 17.17	6/8	
19	Haruyama 2017 [14]	Japan	RCT	Subacute stroke	CSE	Core stabilization exercises	20 min/day, 5times/week, 4 weeks	16	67.56 ± 10.11	3/13	TUG
					CT	Conventional therapy	4 weeks	16	65.63 ± 11.97	4/12	
20	Xie 2018 [57]	China	RCT	Stroke	PME	Tai Chi Yun shou	60 min/day, 5times/week, 12 weeks	120	60.9 ± 8.7	37/83	BBS TUG
					CT	Conventional therapy	12 weeks	124	60.1 ± 8.6	25/99	
21	Knox 2018 [58]	South Africa	RCT	Stroke	RT	Strength intervention	three sets of 10 repetitions, 12 weeks	45	51 ± 12	20/25	BBS TUG
					CT	Conventional therapy	12 weeks	48	48 ± 14	26/22	
22	Krawcyk 2019 [59]	Denmark	RCT	Lacunar stroke	HIIT	HIIT	3 × 3 min with 2 min of active recovery, 5 days/week, 12 weeks	31	63.7 ± 8.9	8/23	MoCA
					CT	Conventional therapy	12 weeks	32	63.7 ± 9.2	6/26	
23	Koch 2020 [60]	America	RCT	Stroke	AE + RT	Combined aerobic and resistance training	50%−65HRmax, 40-60 min/day, 5 consecutive days, 3times/week, 12 weeks	86	59 ± 11	26/60	TUG MoCA
					CT	Conventional therapy	12 weeks	45	58 ± 12	24/21	
24	Yu 2020 [61]	China	RCT	Stroke with hemiplegia	PME	Body weight support-Tai Chi footwork training	40 min/day, 3times/week, 12 week	35	63.03 ± 8.92	14/21	BBS
					CT	Conventional therapy	12 weeks	36	58.69 ± 9.72	16/20	
25	Zheng 2020 [62]	China	RCT	Post-stroke cognitive impairment	PME	Baduanjin	40 min/day, 3times/week, 24 weeks	24	61.63 ± 9.21	5/19	MoCA
					CT	Conventional therapy	24 weeks	24	62.75 ± 6.41	2/22	
26	Chen 2020 [63]	China	RCT	Stroke patients with hemiplegia	CSE	Core muscle training (performed using a multi-point multi-axis suspension training system)	40 min/day, 5-6times/weeks, 8 weeks	90	59.12 ± 12.67	33/57	BBS
					CT	Conventional therapy	8 weeks	90	59.05 ± 12.74	35/53	
27	Min 2020 [64]	Korea	RCT	Chronic stroke	CSE	Trunk stability robot training	30 min/day, 5times/weeks, 4 weeks	19	61.47 ± 11.15	8/11	BBS TUG
					CT	Conventional therapy	4 weeks	19	56.36 ± 9.16	6/13	
28	Soh 2020 [65]	Korea	RCT	Stroke	HIIT	Skater exercise for high-intensity interval training	10 repetitions of the skater exercise and 1 min of light stretching exercise, 30 min/day, 3times/week, 12 week	18	56.3 ± 5.3	5/13	BBS
					AE	Treadmill training	50% to 80% HR reserve, 30 min/day, 3times/week, 12 week	18	57.4 ± 7.2	6/12	
29	da Rosa Pinheiro 2021 [66]	Brazil	RCT	Acute stroke	AE + RT	aerobic cycling training(resistance and no resistance)	50%−70HRmax, 20 min/day, 5 consecutive days	10	63.5 ± 4.5	5/5	BBS
					CT	Conventional therapy	5 days	10	68.9 ± 8.5	7/3	
30	Huang 2021 [67]	China	RCT	Chronic stroke	AE	lateral stair walking exercise	15 min/day, 1 day/week, 12 week	12	53.67 ± 9.16	3/9	TUG
					CT	Conventional therapy	12 weeks	12	63.33 ± 13.31	3/9	
31	Yuen 2021 [68]	China	RCT	Chronic stroke	PME	Baduanjin	50 min/day, 3times/week, 8 weeks	29	63.1 ± 10.6	14/15	TUG
					RT	Strengthening, Weight shifting	50 min/day, 3times/week, 8 weeks	29	62.0 ± 13.1	15/14	
32	Salgueiro 2022 [69]	Spain	RCT	Chronic stroke	CSE	Home based core-stability exercises	5 days/week, 12 weeks	15	57.27 ± 14.35	5/10	BBS
					CT	Conventional therapy	12 weeks	15	64.53 ± 9.40	5/10	
33	Kim 2022 [70]	Korea	RCT	Subacute Stroke	RT	Resistance trunk training	30 min/time, 5times/week, 8 weeks	10	61.5 ± 8.04	5/5	BBS TUG
					CSE	trunk stabilization exercise	30 min/time, 5times/week, 8 weeks	10	61.7 ± 6.66	6/4	
					CT	Conventional therapy	8 weeks	10	61.6 ± 3.92	5/5	
34	Marzolini 2023 [71]	Canada	RCT	Stroke	HIIT + AE	HIIT combined with MICT	MICT for the first 4 weeks(5times/weeks), HIIT for the next 20 weeks(3times/week)	24	62.8 ± 13.2	4/20	BBS MoCA
					AE	MICT	MICT for 24 weeks(5times/week)	23	60.9 ± 8.4	5/18	
35	Duran 2023 [72]	Turkey	RCT	Ischemic stroke	AE	Anti-gravity treadmill training	30 min/day, 3times/week, 4 weeks	13	54.1 ± 18.9	NA	BBS
					CT	Conventional therapy	4 weeks	13	56.1 ± 18.4	NA	
36	Shao 2023 [73]	China	RCT	Stroke	RT	Strength training	45 min/day, 5 days/week, 6 weeks	63	64.56 ± 7.08	22/41	BBS
					CT	Conventional therapy	6 weeks	64	65.72 ± 5.95	21/43	
37	Huang 2024 [74]	China	RCT	Stroke with mild cognitive impairment	AE + RT	Moderate-intensity aerobic exercise(with resistance)	55%−74HRmax, 30 min/day, 5times/day, 2 weeks	10	63.8 ± 7.5	NA	MoCA
					CT	Conventional therapy	2 weeks	10	58.3 ± 8.1	NA	
38	Simić-Panić 2024 [75]	Serbia	RCT	Acute/subacute stroke	AE	cycling exercise	30 min/day, 6 days/week, 3 weeks	59	65 ± 11.98	28/31	BBS TUG
					CT	Conventional therapy	3 weeks	60	67.34 ± 10.86	31/29	
39	Kaymaz 2024 [76]	Turkey	RCT	Hemiplegic patients with stroke	CSE	Trunk stabilization exercises	5 days/week, 4 weeks	10	61.9 ± 7.4	4/6	BBS
					CT	Conventional therapy	4 weeks	10	62.7 ± 8.2	4/6	
40	Kintrilis 2024 [77]	Greece	RCT	Acute stroke	RT	Strength training program	50%−70%Hrmax, 30 min/day, 3 days/weeks, 12 weeks	30	64.9 ± 10.7	14/16	BBS TUG
					CT	Conventional therapy	12 weeks	30	65.4 ± 13.8	14/16	

### Quality evaluation

**Fig. 2:**
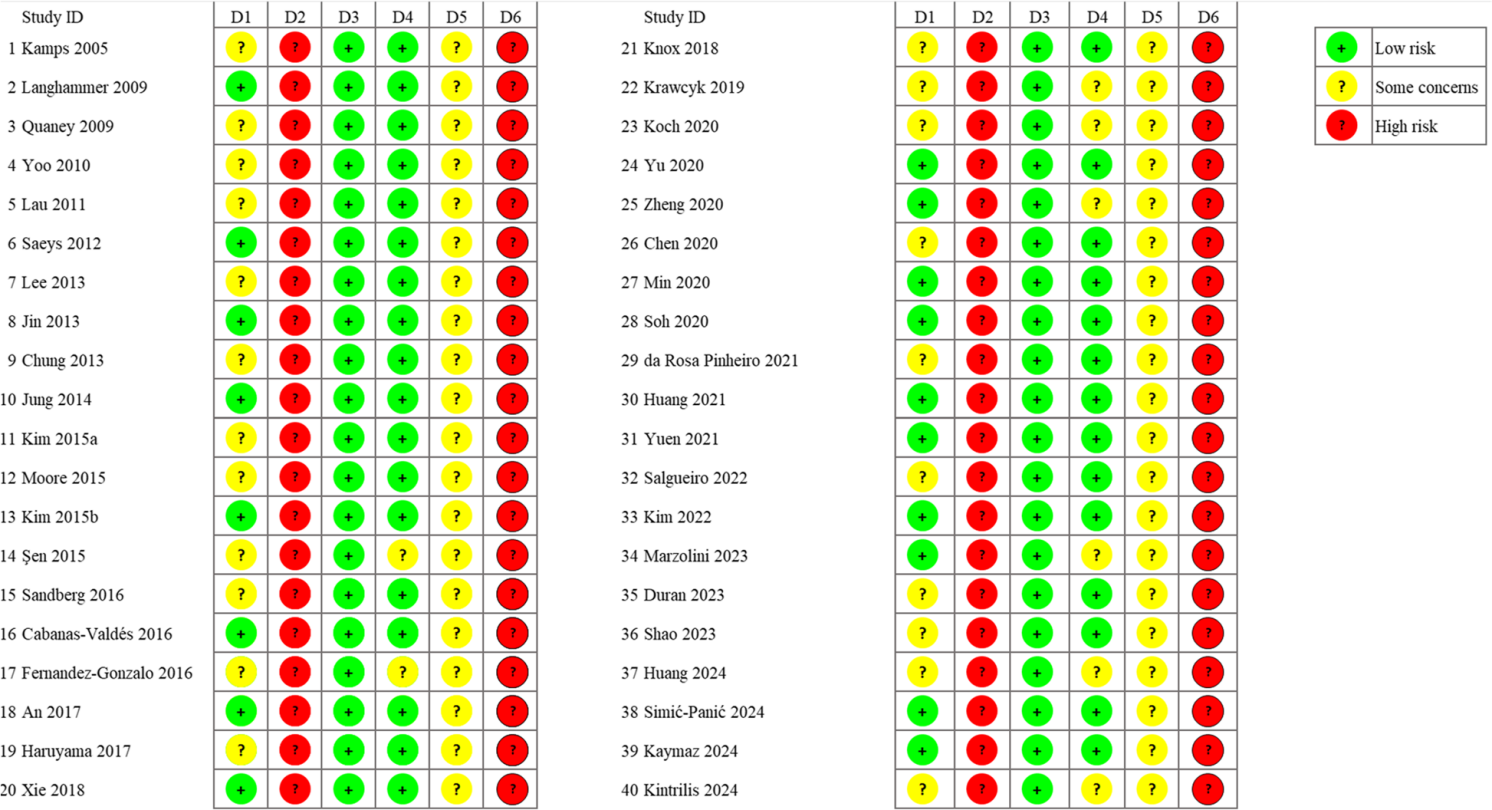
Quality evaluation of each study. Notes: D1, randomization process; D2, deviations from intended interventions; D3, missing outcome data; D4, measurement of the outcome; D5, selection of the reported result; D6, overall

During the evaluation process, two investigators encountered a disagreement regarding the outcome measures of a single article. They collectively reached a final decision by consulting the opinion of a third investigator. The included studies were all RCTs. 18 studies [40, 44, 46, 48, 51, 54, 56, 57, 61, 62, 64, 65, 67, 68, 70, 71, 75, 76] were rated as having a low ROB during the randomization process. 22 studies [14, 39, 41–43, 45, 47, 49, 50, 52, 53, 55, 58–60, 63, 66, 69, 72–74, 77] provided unclear descriptions of the randomization process and were assessed as having some concerns in ROB. Given the particular nature of the exercise interventions, all 40 RCTs may be subject to intervention dependence. Thus, all studies carried a high ROB from deviations in the intended intervention. All studies were judged to have a low ROB related to missing outcome data. 32 studies [14, 39–51, 53, 54, 56–58, 61, 63–70, 72, 73, 75, 76] were judged to have a low ROB in outcome measurement, whereas 8 studies [52, 55, 59, 60, 62, 71, 74, 77] were rated as having some concerns in risk due to the potential awareness of interventions that could influence the measurement outcomes. All studies were appraised as having some concerns in ROB linked to the selective reporting of results (Fig. 2, Table 3).

**Table 3:** Results of literature quality evaluation

ID	Study	Randomization process	Deviations from intended interventions	Missing outcome data	Measurement of the outcome	Selection of the reported result	Overall
1	Kamps 2005	Some concerns	High risk	Low risk	Low risk	Some concerns	High risk
2	Langhammer 2009	Low risk	High risk	Low risk	Low risk	Some concerns	High risk
3	Quaney 2009	Some concerns	High risk	Low risk	Low risk	Some concerns	High risk
4	Yoo 2010	Some concerns	High risk	Low risk	Low risk	Some concerns	High risk
5	Lau 2011	Some concerns	High risk	Low risk	Low risk	Some concerns	High risk
6	Saeys 2012	Low risk	High risk	Low risk	Low risk	Some concerns	High risk
7	Lee 2013	Some concerns	High risk	Low risk	Low risk	Some concerns	High risk
8	Jin 2013	Low risk	High risk	Low risk	Low risk	Some concerns	High risk
9	Chung 2013	Some concerns	High risk	Low risk	Low risk	Some concerns	High risk
10	Jung 2014	Low risk	High risk	Low risk	Low risk	Some concerns	High risk
11	Kim 2015a	Some concerns	High risk	Low risk	Low risk	Some concerns	High risk
12	Moore 2015	Some concerns	High risk	Low risk	Low risk	Some concerns	High risk
13	Kim 2015b	Low risk	High risk	Low risk	Low risk	Some concerns	High risk
14	Şen 2015	Some concerns	High risk	Low risk	Some concerns	Some concerns	High risk
15	Sandberg 2016	Some concerns	High risk	Low risk	Low risk	Some concerns	High risk
16	Cabanas-Valdés 2016	Low risk	High risk	Low risk	Low risk	Some concerns	High risk
17	Fernandez-Gonzalo 2016	Some concerns	High risk	Low risk	Some concerns	Some concerns	High risk
18	An 2017	Low risk	High risk	Low risk	Low risk	Some concerns	High risk
19	Haruyama 2017	Some concerns	High risk	Low risk	Low risk	Some concerns	High risk
20	Xie 2018	Low risk	High risk	Low risk	Low risk	Some concerns	High risk
21	Knox 2018	Some concerns	High risk	Low risk	Low risk	Some concerns	High risk
22	Krawcyk 2019	Some concerns	High risk	Low risk	Some concerns	Some concerns	High risk
23	Koch 2020	Some concerns	High risk	Low risk	Some concerns	Some concerns	High risk
24	Yu 2020	Low risk	High risk	Low risk	Low risk	Some concerns	High risk
25	Zheng 2020	Low risk	High risk	Low risk	Some concerns	Some concerns	High risk
26	Chen 2020	Some concerns	High risk	Low risk	Low risk	Some concerns	High risk
27	Min 2020	Low risk	High risk	Low risk	Low risk	Some concerns	High risk
28	Soh 2020	Low risk	High risk	Low risk	Low risk	Some concerns	High risk
29	da Rosa Pinheiro 2021	Some concerns	High risk	Low risk	Low risk	Some concerns	High risk
30	Huang 2021	Low risk	High risk	Low risk	Low risk	Some concerns	High risk
31	Yuen 2021	Low risk	High risk	Low risk	Low risk	Some concerns	High risk
32	Salgueiro 2022	Some concerns	High risk	Low risk	Low risk	Some concerns	High risk
33	Kim 2022	Low risk	High risk	Low risk	Low risk	Some concerns	High risk
34	Marzolini 2023	Low risk	High risk	Low risk	Some concerns	Some concerns	High risk
35	Duran 2023	Some concerns	High risk	Low risk	Low risk	Some concerns	High risk
36	Shao 2023	Some concerns	High risk	Low risk	Low risk	Some concerns	High risk
37	Huang 2024	Some concerns	High risk	Low risk	Some concerns	Some concerns	High risk
38	Simić-Panić 2024	Low risk	High risk	Low risk	Low risk	Some concerns	High risk
39	Kaymaz 2024	Low risk	High risk	Low risk	Low risk	Some concerns	High risk
40	Kintrilis 2024	Some concerns	High risk	Low risk	Some concerns	Some concerns	High risk

### NMA results

#### BBS

**Fig. 3:**
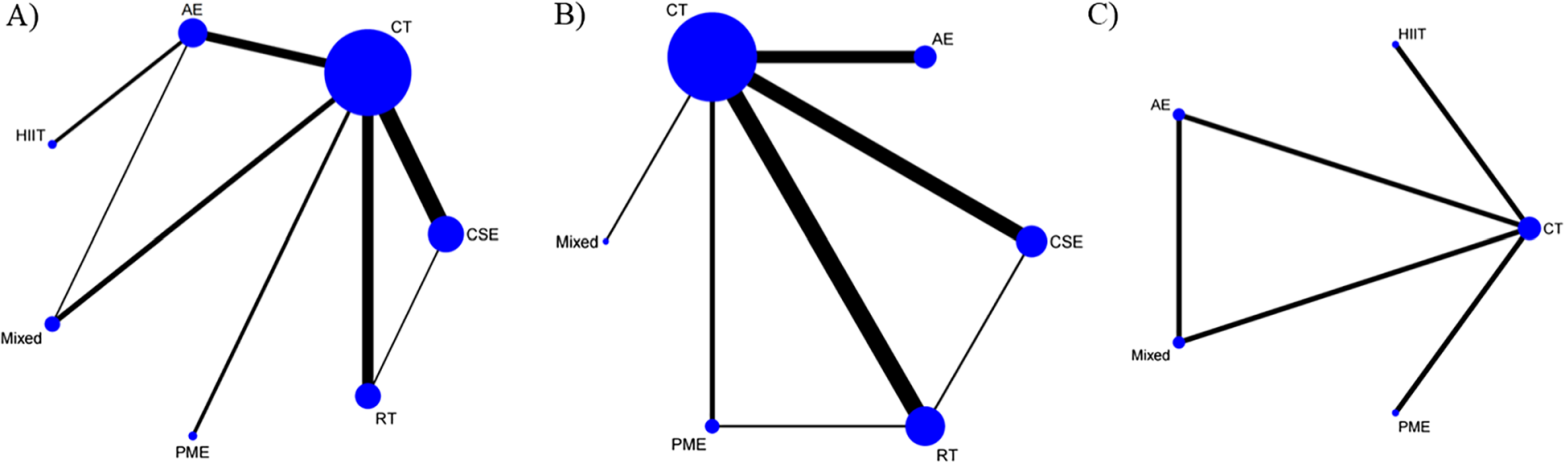
Network plot of the effectiveness of each intervention on BBS, TUG, and MOCA. A) BBS; B) TUG; C) MoCA. The points in the figure represent various interventions, with the size of each point indicating the sample size. The lines connecting two points illustrate direct comparisons between different interventions, where a thicker line signifies a greater number of corresponding studies

27 studies [39, 41–44, 46, 50–52, 54–58, 61, 63–66, 69–73, 75–77] reported on the BBS indicator, encompassing 1,712 patients. Seven interventions were involved, including AE, Mixed, CSE, PME, HIIT, RT, and CT. Figure 3A displays the network plot for the BBS indicator. The points in the figure represent various interventions, with the size of each point indicating the sample size. The lines connecting two points illustrate direct comparisons between different interventions, where a thicker line signifies a greater number of corresponding studies. An inconsistency model was leveraged to assess the overall inconsistency. The difference in DIC was 0.22, suggesting a minor difference. Therefore, the inconsistency was not notable, and a consistency model was selected.

**Fig. 4:**
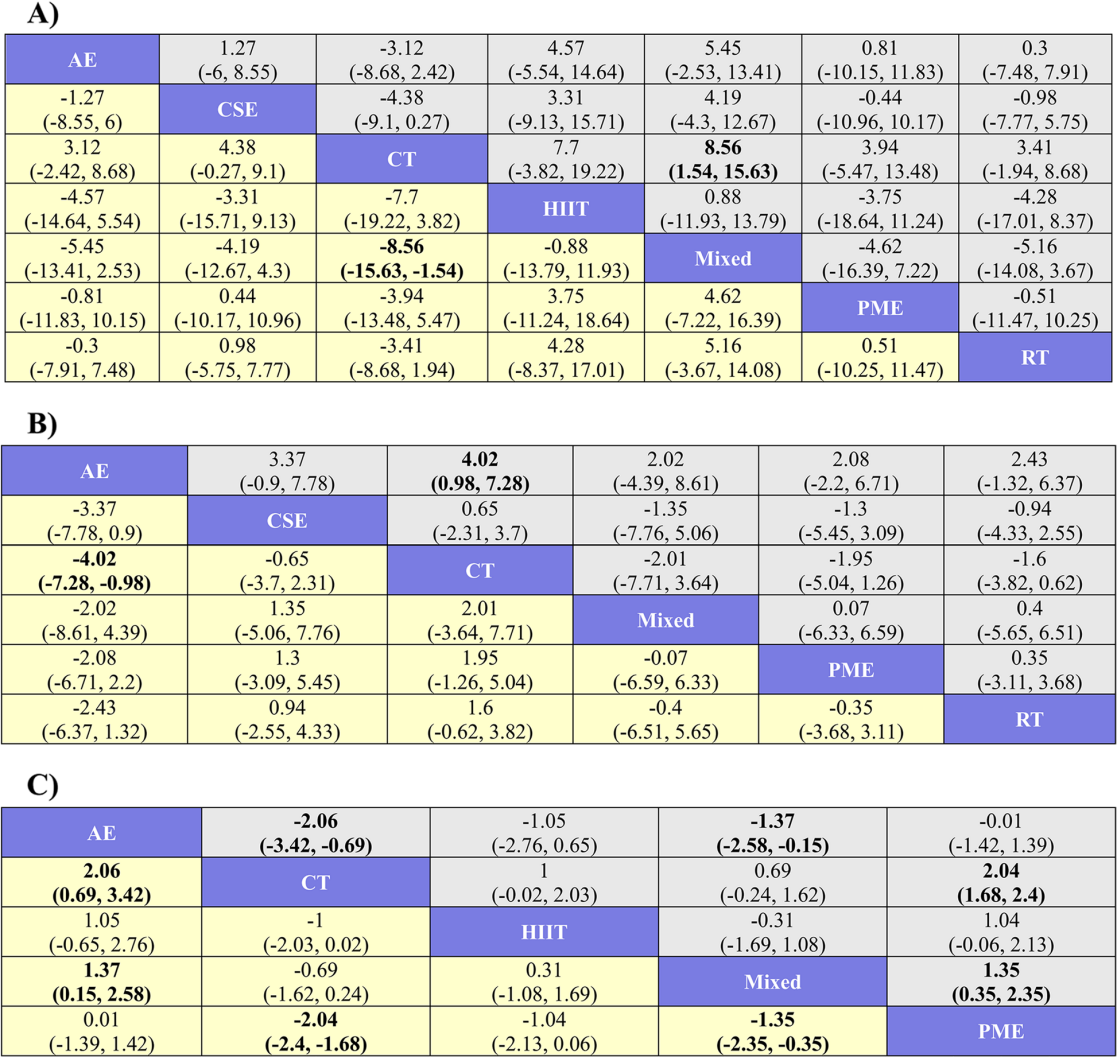
League tables for each outcome indicator. A) BBS; B) TUG; C) MOCA

**Fig. 5:**
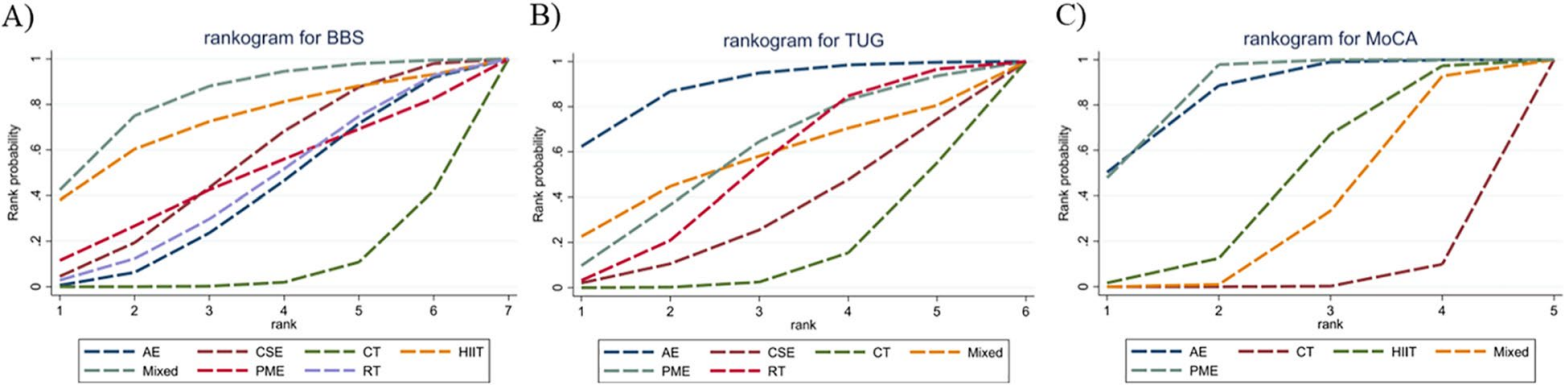
SUCRAs for each outcome indicator. A) BBS; B) TUG; C) MOCA

In the NMA, 21 comparisons were totally established. In relation to CT, Mixed remarkably improved the BBS scores of stroke patients (MD = 8.56, 95% CrI [1.54, 15.63]). The remaining groups exhibited no notable differences among them (Fig. 4A). According to the SUCRA, the ranking of interventions for improving the BBS indicator of patients was as follows: Mixed (82.89%) > HIIT (72.22%) > CSE (53.53%) > PME (48.04%) > RT (44.02%) > AE (40.09%) > CT (9.20%) (Fig. 5A).

The local inconsistency verification was implemented by means of the node splitting method. The results implied that there were no noticeable differences between the direct comparison results and the indirect comparison results for AE vs CT, Mixed vs AE, RT vs CSE, and Mixed vs CT (Figure S1).

#### TUG

21 studies [14, 39, 40, 45, 47–49, 51–53, 55–58, 60, 64, 67, 68, 70, 75, 77] examined the TUG indicator, including 1,206 patients. Six interventions were involved, including AE, Mixed, CSE, RT, PME, and CT. Figure 3B illustrates the network plot for the TUG indicator. The points in the figure represent various interventions, with the size of each point indicating the sample size. The connections between points represent direct comparisons of different interventions, with thicker lines denoting a greater number of studies supporting each comparison. To measure the overall inconsistency, an inconsistency model was employed. The difference in DIC was 0.92, suggesting a minor difference. Therefore, the inconsistency was not notable, and a consistency model was selected.

The NMA revealed that a total of 15 comparisons were generated. Compared to CT, AE noticeably reduced TUG indicator (MD = −4.02, 95% CrI [−7.28, −0.98]). No other comparisons exhibited statistically significant differences (Fig. 4B). According to the SUCRA, the ranking of interventions for improving the TUG indicator was as follows: AE (88.46%) > PME (57.56%) > Mixed (55.31%) > RT (51.96%) > CSE (32.06%) > CT (14.66%) (Fig. 5B).

According to the local inconsistency verification, no noticeable differences were noted between the direct comparison results of RT vs CSE, PME vs CT, RT vs CT, and RT vs PME and their respective indirect comparison results (Figure S2).

##### MoCA

Five studies [59, 60, 62, 71, 74] reported the MoCA indicator, with 309 patients. Five interventions were involved, including AE, Mixed, HIIT, PME, and CT. The network plot for the MoCA indicator is presented in Fig. 3C. In this plot, each point represents a different intervention. The size of the point corresponds to the sample size, and the lines connecting points indicate direct comparisons between interventions. Thicker lines correspond to a greater number of studies supporting those comparisons. Overall inconsistency was assessed using an inconsistency model, resulting in a DIC difference of 0.96, which suggested a negligible inconsistency. As a result, a consistency model was selected.

The results of the NMA indicated that a total of 10 comparisons were generated. Compared to CT, AE (MD = 2.06, 95% CrI [0.69, 3.42]) and PME (MD = 2.04, 95% CrI [1.68, 2.4]) exhibited superior effects on improving patients’MoCA scores. Additionally, AE (MD = 1.37, 95% CrI [0.15, 2.58]) and PME (MD = 1.35, 95% CrI [0.35, 2.35]) outperformed Mixed. The remaining groups exhibited no noticeable differences among them (Fig. 4C). Based on the SUCRA, the ranking of interventions for improving the MoCA indicator was as follows: PME (86.43%) > AE (84.46%) > HIIT (44.70%) > Mixed (31.83%) > CT (2.57%) (Fig. 5C).

The local inconsistency test using the node splitting method showed no notable differences between direct and indirect comparisons of CT vs AE, Mixed vs AE, and Mixed vs CT (Figure S3).

### Subgroup analysis

Considering the potential impact of intervention duration on outcomes, we executed a subgroup analysis of BBS and TUG metrics based on intervention time. The intervention time was split into two categories: under 12 weeks and 12 weeks or longer. Due to the insufficient number of original studies for the MoCA outcome measure, subgroup analysis was not conducted on the MoCA indicator.

The subgroup analysis results for the BBS score indicated that there were 16 studies with intervention durations of less than 12 weeks [41–44, 51, 52, 54, 56, 63, 64, 66, 70, 72, 73, 75, 76], involving 907 participants. Six interventions were involved, including AE, CSE, RT, Mixed, HIIT, and CT (Figure S4A). The DIC difference of 0.09 suggested low inconsistency, and a consistency model was applied for the analysis. The NMA resulted in a total of 15 comparisons. Compared to CT, Mixed noticeably improved the BBS scores of stroke patients (MD = 16.07, 95% CrI [4.7, 26.51]), while no evident differences were observed in other comparisons (Figure S5A). Based on the SUCRA, the ranking of interventions for improving the BBS scores was as follows: Mixed (94.50%) > RT (58.40%) > CSE (53.25%) > HIIT (47.16%) > AE (33.22%) > CT (13.47%). (Figure S6A).

11 studies with intervention durations of 12 weeks or longer included 805 participants [39, 46, 50, 55, 57, 58, 61, 65, 69, 71, 77]. Seven interventions were involved, including AE, CSE, RT, Mixed, HIIT, PME, and CT (Figure S7A). A DIC difference of 0.13 indicated minimal inconsistency, so a consistency model was used for analysis. The NMA resulted in 21 comparisons, all of which exhibited no remarkable differences (Figure S8A). Based on the SUCRA, the ranking of interventions for improving the BBS scores was as follows: HIIT (80.89%) > PME (63.10%) > Mixed (47.80%) > AE (46.20%) > CSE (41.13%) > RT (40.11%) > CT (30.78%). (Figure S9A).

The subgroup analysis results for the TUG indicator demonstrated that there were 12 studies with an intervention duration of less than 12 weeks [14, 45, 47–49, 51, 52, 56, 64, 68, 70, 75], involving 511 participants. Five interventions were involved, including AE, PME, RT, CSE, and CT. (Figure S4B). The DIC difference of 0.79 reflected a small inconsistency, thus a consistency model was leveraged in the analysis. The NMA resulted in 10 comparisons. In comparison with CT, AE (MD = −5.16, 95% CrI [−11.01, −0.32]) and RT (MD = −3.63, 95% CrI [−6.91, −0.06]) apparently lowered the TUG indicator for stroke patients. In contrast, the remaining comparisons did not show any marked differences (Figure S5B). Based on the SUCRA, the ranking of interventions for improving the TUG indicator was as follows: AE (81.39%) > PME (71.84%) > RT (63.69%) > CSE (26.42%) > CT (6.68%). (Figure S6B).

There are 9 studies with an intervention duration of 12 weeks or more, involving 743 participants [39, 40, 53, 55, 57, 58, 60, 67, 77]. Five interventions were involved, including AE, RT, Mixed, PME, and CT (Figure S7B). A DIC difference of 0.02 demonstrated a small inconsistency, suggesting the use of a consistency model for analysis. The NMA generated 10 comparisons, all of which showed no noticeable differences (Figure S8B). Based on the SUCRA, the ranking of interventions for improving the TUG indicator was as follows: AE (85.72%) > Mixed (66.31%) > RT (40.01%) > CT (34.78%) > PME (23.18%). (Figure S9B).

### Publication bias analysis

**Fig. 6:**
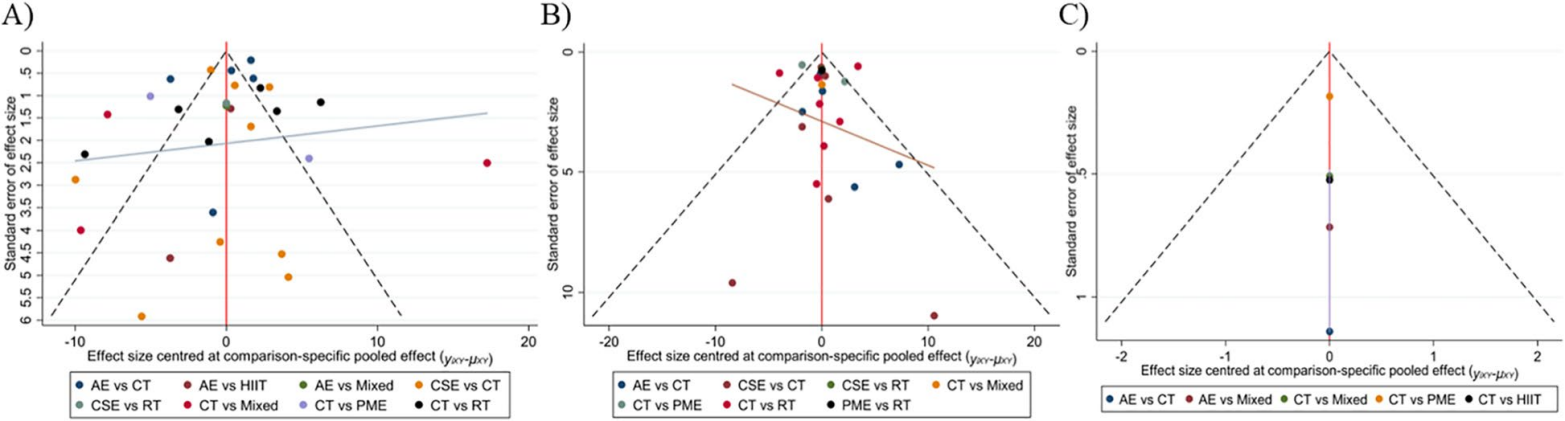
Publication bias for each outcome indicator. A) BBS; B) TUG; C) MOCA

In the funnel plot illustrating publication bias, each colored dot corresponded to a pairwise comparison between different interventions (Fig. 6). The number of dots increased with the frequency of pairwise comparisons. The symmetrical distribution of points in the funnel plot suggested a low risk of publication bias. As shown in Figs. 6A, 6B, and 6 C, the points were symmetrically distributed in the plots, indicating minimal publication bias for the BBS, TUG, and MoCA metrics. However, given the limited number of original studies on MoCA measures, the funnel plot's effect estimates may be subject to bias and these results should therefore be interpreted cautiously. Due to the involvement of more than ten articles in both the BBS and TUG metrics, we conducted an Egger’s test to appraise publication bias. The results of the Egger test indicated that the p-value for the BBS score was 0.308, while the p-value for the TUG indicator was 0.483. These findings further confirmed that there was no publication bias linked to either the BBS or TUG metrics.

### Evidence quality assessment

The quality of evidence for this NMA was appraised by means of the GRADE approach. Due to the unique nature of exercise interventions, blinding was often not feasible. Furthermore, most studies typically did not disclose their protocols or statistical analysis methods, which contributed to a higher ROB in the included studies. In light of this, we had appropriately lowered our evaluation standards. In the within-study bias section, concerns categorized as"some concerns"remained unchanged, while those classified as"major concerns"were downgraded by one level. The GRADE results indicated that among the 21 comparisons for BBS, 1 had evidence quality rated as low, while 20 were rated as very low (Table S1). In the 15 comparisons for TUG, the quality of evidence for 1 comparison was rated as moderate, 1 as low, and 13 as very low (Table S2). In the 10 comparisons for MoCA, 3 exhibited evidence quality rated as moderate, 2 demonstrated evidence quality classified as low, and 5 were assessed with very low evidence quality (Table S3). The overall quality of evidence in this NMA was relatively low, and caution should be exercised when interpreting the results.

## Discussion

### Main findings and clinical significance

The present NMA ultimately includes 40 studies. According to the SUCRA results, Mixed is identified as the most effective approach for improving BBS scores in stroke patients. Additionally, HIIT demonstrates a high efficacy in enhancing the BBS score. CSE, PME, RT, and AE exhibit comparable effectiveness in improving BBS scores as well. AE is the most effective intervention for improving the TUG indicator in stroke patients, while PME, Mixed, RT, and CSE demonstrate comparable efficacy in enhancing TUG performance. PME and AE are the most effective interventions for improving the MoCA indicator, with HIIT and AE demonstrating comparable efficacy in enhancing the MoCA indicator. These results are influenced by various factors, including the timing of exercise interventions and the patient’s stage of recovery. Therefore, when formulating exercise prescriptions for stroke patients, it is essential to consider a comprehensive approach that takes into account the patient’s treatment objectives and recovery phase.

### Comparative efficacy of different exercises on balance function improvement

The BBS and TUG are commonly utilized assessment tools for evaluating balance function in the clinical treatment of stroke patients. The BBS is relatively comprehensive and integrates both dynamic balance and static balance functional components [78]. The TUG test primarily serves to assess the dynamic balance of patients during functional mobility [79]. The selection of the BBS and TUG as the primary tools for assessing balance function is due to their widespread application in clinical rehabilitation therapy [80]. Compared to other balance assessment tools like the Fugl-Meyer balance scale, the sensory organization test, the functional gait assessment, walking speed, and the functional reach test (FRT), both the BBS and TUG test are extensively employed in studies [81]. This greater availability of studies facilitates their inclusion in NMA, thereby enhancing statistical power. The FRT and walking speed are not as comprehensive as the BBS and TUG in assessing balance function. Moreover, the BBS and TUG demonstrate a higher level of standardization compared to tools such as the Tinetti test [82, 83]. Their reduced reliance on subjective scoring contributes to minimizing measurement bias, thereby decreasing heterogeneity across studies. Additionally, due to the particular considerations for NMA, most studies simultaneously report the BBS and TUG, making it easier to build a coherent network structure and increase node consistency.

Regarding the improvement of BBS, noticeable effects are observed only in the comparison between Mixed and CT, with no evident differences in the other comparisons. According to the SUCRA results, Mixed is identified as the most effective approach for improving BBS scores in stroke patients. Notably, Mixed improved BBS by about 8.5 points. An 8.5-point increase in BBS exceeds the minimal clinically important difference (MCID) of 5 points, indicating a significantly enhanced postural stability and reduced risk of falls. Additionally, HIIT demonstrates a high efficacy in enhancing the BBS score. CSE, PME, RT, and AE exhibit comparable effectiveness in improving BBS scores as well. Five studies included in the research report the efficacy of Mixed for BBS. The primary components of Mixed include AE, RT, and HIIT. Based on previous research, Mixed has demonstrated noticeable advantages across various populations [84, 85], effectively serving as a complementary approach that enhances the strengths of different forms of exercise. RT can enhance the muscle strength of anti-gravity muscle groups, like the quadriceps and gluteal muscles in patients [86]. Additionally, certain forms of bilateral symmetrical RT can address the issue of muscular strength imbalance on the affected side in individuals with hemiplegia [87]. The characteristics of HIIT, which involves short-duration, high-load stimuli, are more conducive to enhancing neuromuscular response speed and strengthening the ability to transfer the center of gravity [88, 89]. The relatively low intensity of AE with a longer duration is beneficial for enhancing muscle endurance, prolonging balance time, and reducing the phenomenon of fatigue-related imbalance during physical activity [90]. This makes AE a valuable complement to RT and HIIT. In clinical treatment, when patients exhibit relatively strong physical functions and motor abilities, particularly in the chronic phase, Mixed may be prioritized [91, 92]. This method combines various exercise interventions to leverage the compensatory effects of different types of exercises. The standalone HIIT possesses the characteristic of providing short bursts of high-intensity stimulation. However, its effectiveness is diminished compared to Mixed due to the absence of endurance exercises that complement muscle endurance and control over exercise rhythm [93]. The effects of CSE, PME, RT, and AE on improving BBS scores are comparable. This further suggests that merely increasing muscle strength may be insufficient to significantly enhance balance function. Instead, it is necessary to incorporate neuromuscular control or employ higher-intensity exercise stimuli as an adjunct [94, 95]. The results of the subgroup analysis indicate that when the intervention duration is less than 12 weeks, Mixed is the most effective intervention. The efficacy of RT, CSE, HIIT, and AE is comparable. Conversely, when the intervention duration is greater than or equal to 12 weeks, HIIT emerges as the most effective intervention, while PME, Mixed, AE, CSE, and RT demonstrate similar efficacy. The results of the subgroup analysis may be influenced to some extent by the number of studies. However, this further suggests that high-intensity exercise stimuli, combined with the consolidation of long-term physical activity, may be more beneficial for enhancing neuromuscular control and improving balance function.

In terms of improving dynamic balance ability as measured by the TUG test, only the comparison between AE and CT demonstrated a significant effect, while no other comparisons showed statistically significant differences. The SUCRA results indicate that AE is the most effective intervention for improving TUG, while PME, Mixed, RT, and CSE demonstrate comparable efficacy in enhancing TUG performance. The TUG test primarily evaluates the continuous actions of rising, walking, turning, and sitting down, which necessitates certain levels of cardiovascular endurance, gait efficiency, and dynamic postural adjustment capabilities [96, 97]. The direct effects of AE include the enhancement of patients’cardiopulmonary function, increased muscular endurance, and improved rhythm control during physical activity [11, 90]. In this NMA, AE reduced TUG by 4 s. A reduction of 4 s in TUG likely shifts patients from limited community ambulation (> 13.5 s) to a safer mobility category, possibly improving their independence in ADLs. Despite this, standalone AE may lack the foundational benefits of RT in enhancing muscle strength, as well as the stimulating effects of HIIT on neuromuscular control. Therefore, combining AE with RT and HIIT in a mixed approach may yield more favorable outcomes. However, in this NMA, only two studies employed Mixed as an intervention. The limited number of studies may result in insufficient statistical power, leading to a non-significant effect of Mixed and a ranking lower than that of AE. PME, such as Tai Chi and Ba Duan Jin, can enhance the perception and control of weight transfer [98, 99], thereby improving the stability of joints and muscles during movement [100]. However, these activities present a higher level of difficulty and require significant physical function and motor capabilities from patients. Therefore, they are primarily suitable for stroke patients in the chronic phase [101]. In the chronic phase, combining PME with training methods like RT and CSE can effectively enhance muscle strength while also improving movement perception and postural control abilities. This integrated approach lays a solid foundation for enhancing dynamic balance in patients. The results of the subgroup analysis indicate that both short-term interventions (less than 12 weeks) and long-term interventions (greater than or equal to 12 weeks) demonstrate that AE is the most effective intervention measure. This indicates that the effects of AE on enhancing cardiovascular endurance and improving neuromuscular adaptability are less influenced by the duration of intervention. This result further corroborates the theoretical frameworks established by Ferrari et al*.* (2013) [102] and Oorschot et al*.* (2023) [103].

### Comparative efficacy of different exercises on cognitive function improvement

In the assessment of cognitive function in stroke patients, we prioritized the MoCA as our evaluation tool. This choice is based on MoCA’s widespread use in clinical settings for assessing cognitive function in stroke patients and its comprehensive coverage of key domains such as executive function, memory, language, and visuospatial skills [104, 105]. An NMA necessitates a high degree of consistency in outcome measures across studies to ensure comparability of effect sizes. Thus, the standardized scoring system of MoCA is particularly well-suited for conducting NMA. While other tools such as the WCST, MMSE, and TMT are also effective for assessing cognitive function, these instruments tend to focus on a single dimension of cognition and have been reported less frequently in included RCTs. Furthermore, they involve fewer intervention measures. Considering the statistical power and network connectivity required for NMA alongside the need for a comprehensive evaluation of cognitive function, we ultimately chose MoCA as our primary assessment tool.

Previous studies have confirmed the positive effects of exercise on improving cognitive functions (such as memory, executive function, and attention) in stroke patients [106]. Nevertheless, there is still considerable debate surrounding this field of research. The controversies primarily revolve around skepticism regarding therapeutic effects, differing interpretations of exercise modalities, and discussions on the impact of varying intervention intensities. This NMA identifies PME as the most effective intervention for improving cognitive function in stroke patients. This finding further indicates that exercise combined with cognitive components may be more beneficial than exercise alone [107]. Moreover, compared to conventional treatment, PME demonstrates a more pronounced therapeutic effect on cognitive function. The remarkable impact of PME on cognitive function improvement may stem from its ability to promote concentration, alleviate mental stress, and enhance mind–body coordination. From a physiological perspective, PME can enhance the oxygen content in the brain and activate neuronal activity. The enhanced oxygen delivery and neural excitability boost the patient’s responsiveness and cognitive ability, facilitating the recovery of cognitive function [108]. One study also suggests that PME like Baduanjin helps modulate the functional connectivity of the locus coeruleus and ventral tegmental area, increasing the activity of the norepinephrine and dopamine systems. These improvements are essential for cognitive function restoration, thereby improving patients’attention and memory capabilities [109]. In clinical treatment, compared to other exercise interventions, PME stands out due to its low intensity and high level of enjoyment. Current studies have confirmed that PME demonstrates higher safety and fewer side effects in the rehabilitation of chronic diseases [110, 111]. Furthermore, the engaging nature of PME makes it more suitable for stroke patients, thereby enhancing compliance and improving the long-term effectiveness of the intervention [112]. In the population of patients with cognitive dysfunction following a stroke, the characteristics of PME integrating movement, balance, and respiratory control while simultaneously engaging attention, working memory, and executive functions render it the most suitable intervention.

Earlier studies have also debated whether high-intensity exercise or low-to-moderate-intensity exercise is more effective [19, 113]. The results of the NMA indicate that AE demonstrates a strong efficacy in improving cognitive function, comparable to PME. In contrast, HIIT and Mixed show relatively lower effectiveness in enhancing cognitive function. This suggests that moderate-intensity, prolonged stimulation may be more beneficial for the stable promotion of BDNF secretion, whereas high-intensity exercise may lead to higher peak levels of BDNF but with a shorter duration of maintenance. Additionally, the high intensity and brief duration of HIIT may lead to cognitive overload in some patients, which limits their potential for cognitive improvement [114]. For stroke patients with cognitive impairments, it is important to cautiously choose high-intensity training to prevent unnecessary psychological strain and physical stress. The drawbacks of high-intensity exercise training on cognitive function improvement are more pronounced in Mixed. The primary components of Mixed in the NMA for the MoCA indicator are the combination of AE and HIIT. Both AE and HIIT are highly demanding rehabilitation strategies for stroke patients. Combining the two further increases training duration and intensity, making fatigue particularly likely. This is especially relevant for stroke patients, whose motor deficits may lead to heightened neural stress during exercise, significantly increasing the risk of fatigue and potentially offsetting some post-exercise benefits [115, 116]. Therefore, for stroke patients whose primary goal is to improve cognitive function, it is essential to exercise caution when selecting high-intensity intervention measures. Preference should be given to moderate-intensity exercises with longer durations.

### Clinical implications and exercise prescription recommendations

This NMA found that Mixed and AE were significantly effective for improving balance function, and PME and Mixed were significantly effective for enhancing cognitive function. Although our study focused on balance and cognitive function, it also laid the foundation for achieving broader rehabilitation goals. The improvement of balance function in stroke patients is closely related to their fall risk and mobility [117, 118], and the improvement of cognitive function is closely related to social participation [119]. Overall, improving balance and cognitive functions is conducive to achieving comprehensive rehabilitation goals.

Based on the findings of this NMA, we recommend selecting types of exercise tailored to the diverse needs of stroke patients. For stroke patients with abnormal balance ability, gait abnormalities, and a high risk of falls, it is recommended to prioritize the use of Mixed to enhance balance function. Specifically, it is recommended to employ RT and CSE to enhance muscle strength. Additionally, moderate implementation of HIIT should be utilized for intense stimulation, followed by the incorporation of AE for consolidation. When patients exhibit weakened motor function or physical capabilities, particularly in the acute or subacute phases of stroke, Mixed is often challenging to implement. Therefore, it is advisable to initially adopt a singular mode of exercise. The selection of alternative approaches such as RT and CSE should be tailored to the individual characteristics of the patient. When necessary, therapists should proactively provide assistance to help patients enhance their muscle strength, thereby laying a solid foundation for subsequent mixed interventions.

For patients with cognitive function impairments, the restoration of cognitive abilities is a primary need. Therefore, it is recommended to prioritize PME or AE as the main intervention measures. If the patient’s physical and motor functions are relatively strong, PME should be prioritized. This is because PME can effectively address both exercise and the social functioning of the patient, while its engaging nature promotes long-term adherence to the program. Furthermore, in this population of patients, caution should be exercised when selecting high-intensity exercises. Moderate intensity is recommended instead, along with prolonged intervention periods to ensure maintenance of benefits.

### Evidence quality assessment

We leveraged the GRADE quality assessment tool to appraise the quality of evidence for each comparison. The results indicate that the overall quality of evidence for this NMA is relatively low. Firstly, the intervention of exercise possesses certain specificity, making it difficult to implement blinding in most studies. Additionally, many studies typically do not disclose their protocols or statistical analysis methods, which hampers our ability to determine whether there is bias in the selective reporting of results. These factors contribute to a higher ROB in the included studies. In this NMA, the heterogeneity among studies is significantly influenced by various factors, including differences in patient age, stroke recovery stages, types of strokes, and specific implementation plans for interventions and control measures. Consequently, there exists a certain risk of inconsistency in the final results. Moreover, there are no direct comparisons between certain intervention measures, which rely on indirect evidence from networks. This reliance has resulted in a significant degree of indirectness in some of the evidence. In cases where the quality of evidence is relatively low, the SUCRA rankings may be unstable. Therefore, it is essential to interpret the results of this NMA with caution. Additionally, when formulating exercise prescriptions, clinical practicalities should also be taken into account for informed decision-making.

### Methodological limitations

This study has several limitations. Firstly, the sample size of the included studies is limited, and some interventions are conducted over a relatively short duration, which may affect the stability and generalizability of the results. Although we executed a subgroup analysis based on the timing of interventions, the stability of the results may be influenced to some extent by the limited number of studies. Secondly, although some studies adopted the same intervention measures, notable differences still exist in aspects such as the intensity, frequency, and environment of the exercise interventions. These discrepancies may lead to significant heterogeneity, thereby affecting the accuracy of the results. In addition, Mixed in our study mainly consists of AE and RT or comprises HIIT and AE. Different mixed modes may also have different intervention effects. However, due to the small number of studies, we were unable to conduct a sensitivity analysis. Thirdly, regarding cognitive function indicators in stroke patients, there is a lack of high-quality studies, and the methods used to assess cognitive function vary considerably among the available studies. Most studies rely on the MoCA. Other assessment tools, such as the MMSE, TMT, and WCST, are also valuable for evaluating cognitive function in stroke patients. However, due to the limited number of studies using these tools, they could not be included in the NMA to ensure the reliability of the findings. Fourthly, this NMA restricts the inclusion of studies to only English-language publications that have undergone peer review, thereby ensuring a standardized quality assessment. However, this criterion may also lead to the omission of recent yet unverified studies. Fifthly, while some effect sizes, like an increase of 2.04 or 2.06 points in MoCA, are statistically significant, their clinical significance should be prioritized. Caution is warranted during the interpretation of these results. Sixthly, the recovery stages of stroke (including acute, subacute, and chronic phases) may have a certain impact on the results of NMA. However, among the 40 studies ultimately included in the NMA, only 22 report specific recovery stages of stroke, while 18 do not provide this information. Furthermore, some studies that reported on recovery stages included multiple different phases without distinguishing between them during outcome measurement. These issues make it challenging for us to conduct separate analyses and comparisons of stroke patients at different stages of recovery. Seventhly, among the original studies included in the NMA, only a limited number of articles conducted long-term follow-ups. This limitation prevents us from analyzing the long-term effects of different types of exercise on balance and cognitive function in stroke patients within the NMA.

Future studies should further analyze factors such as sex, age, and disease severity, to determine the optimal exercise regimen for different individuals. Moreover, the long-term effects of exercise on stroke patients remain insufficiently explored. Future studies should place greater emphasis on the prolonged impact of exercise on neurological function in stroke patients, thereby providing both short-term intensive recommendations and long-term exercise training programs. Additionally, further investigation into combined intervention strategies, like integrating exercise with pharmacological treatments or cognitive training, is necessary to develop more evidence-based and effective clinical recommendations for patients.

## Conclusion

This NMA examines the effects of various exercise interventions on balance and cognitive function in stroke patients. Mixed and AE noticeably enhance the balance function in stroke patients, while PME and AE notably improve cognitive function in this population. The efficacy of other exercise interventions requires further validation. In patients whose main intervention goal is to improve balance function, we recommend prioritizing the combination of RT, HIIT, and AE to enhance balance function when the patient’s mobility and physical fitness are relatively strong. For patients with poor mobility and physical function, the priority should be the combination of RT and CSE to enhance basic strength. In patients whose primary intervention goal is to enhance cognitive function, we recommend prioritizing the use of PME when the patients exhibit strong motor abilities and physical fitness. Conversely, in cases where patients have relatively poor motor skills and physical capabilities, it is advisable to opt for AE with a lower level of difficulty.

## Supplementary Information

Supplementary Material 1: Appendix 1. PRISMA + NMA + checklist. Appendix 2. Search Strategies. Table S1. GRADE Evidence Results of the BBS Indicator. Table S2. GRADE Evidence Results of the TUG Indicator. Table S3. GRADE Evidence Results of the MoCA Indicator. Figure S1. Node-splitting method results for the BBS indicato. Figure S2. Node-splitting method results for the TUG indicato. Figure S3. Node-splitting method results for the MoCA indicato. Figure S4. Network plot of the effectiveness of each interventionon BBS, TUG, and MoCA Scores. A) BBS; B) TUG. The points in the figure represent various interventions, with the size of each point indicating the sample size. The lines connecting two points illustrate direct comparisons between different interventions, where a thicker line signifies a greater number of corresponding studies. Figure S5. League tables for each outcome indicator in the < 12-week intervention subgroup A) BBS; B) TUG. Figure S6. SUCRAs for each outcome indicator in the < 12-week intervention subgroup. A) BBS; B) TUG. Figure S7. Network plot of the effectiveness of each interventionon BBS, TUG, and MoCA Scores. A) BBS; B) TUG. The points in the figure represent various interventions, with the size of each point indicating the sample size. The lines connecting two points illustrate direct comparisons between different interventions, where a thicker line signifies a greater number of corresponding studies. Figure S8. League tables for each outcome indicator in the ⩾12-week intervention subgroup A) BBS; B) TUG. Figure S9. SUCRAs for each outcome indicator in the ⩾12-week intervention subgroup. A) BBS; B) TUG

## Data Availability

The data that support the findings of this study are available from the corresponding author upon reasonable request.

## Abbreviations

NMA: Network meta-analysis
RCTs: Randomized controlled trialsCSE: core stability exercise
PME: Physical/mental exercise
HIIT: High-intensity interval training
AE: Aerobic exercise
RT: Resistance training
CT: Conventional treatment
BBS: Berg balance scale
TUG: Timed up and go
MoCA: Montreal cognitive assessment
MD: Mean difference
DIC: Deviance information criterion
CrI: Credible interval
WCST: Wisconsin card sorting test
TMT: Trail making test
MMSE: Mini-mental state examination;
Mixed: Mixed-component exercise
