# Supplementary material for: Effectiveness of different exercise interventions on balance and cognitive functions in stroke patients: A network meta-analysis
Source: BMC Sports Sci Med Rehabil. 2025 Aug 27;17:250. doi: 10.1186/s13102-025-01267-3 (PMC12382136; doi:10.1186/s13102-025-01267-3)
Supplement: Supplementary file 1 — Supplementary Material 1: Appendix 1. PRISMA + NMA + checklist. Appendix 2. Search Strategies. Table S1. GRADE Evidence Results of the BBS Indicator. Table S2. GRADE Evidence Results of the TUG Indicator. Table S3. GRADE Evidence Results of the MoCA Indicator. Figure S1. Node-splitting method results for the BBS indicato. Figure S2. Node-splitting method results for the TUG indicato. Figure S3. Node-splitting method results for the MoCA indicato. Figure S4. Network plot of the effectiveness of each interventionon BBS, TUG, and MoCA Scores. A) BBS; B) TUG. The points in the figure represent various interventions, with the size of each point indicating the sample size. The lines connecting two points illustrate direct comparisons between different interventions, where a thicker line signifies a greater number of corresponding studies. Figure S5. League tables for each outcome indicator in the < 12-week intervention subgroup A) BBS; B) TUG. Figure S6. SUCRAs for each outcome indicator in the < 12-week intervention subgroup. A) BBS; B) TUG. Figure S7. Network plot of the effectiveness of each interventionon BBS, TUG, and MoCA Scores. A) BBS; B) TUG. The points in the figure represent various interventions, with the size of each point indicating the sample size. The lines connecting two points illustrate direct comparisons between different interventions, where a thicker line signifies a greater number of corresponding studies. Figure S8. League tables for each outcome indicator in the ⩾12-week intervention subgroup A) BBS; B) TUG. Figure S9. SUCRAs for each outcome indicator in the ⩾12-week intervention subgroup. A) BBS; B) TUG [file 13102_2025_1267_MOESM1_ESM.docx]

**Appendix 1:** PRISMA+NMA+checklist

| **Section/topic** | **#** | **Checklist item** | **Reported on page #** |
| --- | --- | --- | --- |
| **TITLE** | | |  |
| Title | 1 | Identify the report as a systematic review incorporating a network meta-analysis (or related form of  meta-analysis). | 1 |
| **ABSTRACT** | | |  |
| Structured summary | 2 | Provide a structured summary including, as applicable:  Background: main objectives  Methods: data sources; study eligibility criteria, participants, and interventions; study appraisal; and synthesis methods, such as network meta-analysis.  Results: number of studies and participants identified; summary estimates with corresponding confidence/credible intervals; treatment rankings may also be discussed. Authors may choose to summarize pairwise comparisons against a chosen treatment included in their analyses for brevity.  Discussion/Conclusions: limitations; conclusions and implications of findings.  Other: primary source of funding; systematic review registration number with registry name. | 1-2 |
| **INTRODUCTION** | | |  |
| Rationale | 3 | Describe the rationale for the review in the context of what is already known, including mention of why a network meta-analysis has been conducted | 2-5 |
| Objectives | 4 | Provide an explicit statement of questions being addressed with reference to participants, interventions, comparisons, outcomes, and study design (PICOS). | 4-5 |
| **METHODS** | | |  |
| Protocol and registration | 5 | Indicate if a review protocol exists and if and where it can be accessed (e.g., Web address), and, if available, provide registration information including registration number. | 6 |
| Eligibility criteria | 6 | Specify study characteristics (e.g., PICOS, length of follow-up) and report characteristics (e.g., years considered, language, publication status) used as criteria for eligibility, giving rationale. Clearly describe eligible treatments included in the treatment network and note whether any have been clustered or merged into the same node (with justification). | 6 |
| Information sources | 7 | Describe all information sources (e.g., databases with dates of coverage, contact with study authors to identify additional studies) in the search and date last searched. | 6 |
| Search | 8 | Present full electronic search strategies for at least one database, including any limits used, such that it could be repeated. | 7 |
| Study selection | 9 | State the process for selecting studies (i.e., screening, eligibility, included in systematic reviews, and, if applicable, included in the meta-analysis). | 7 |
| Data collection process | 10 | Describe the method of data extraction from reports (e.g., piloted forms, independently, in duplicate) and any processes for obtaining and confirming data from investigators. | 8 |
| Data items | 11 | List and define all variables for which data were sought (e.g., PICOS, funding sources) and any assumptions and simplifications made. | 8 |
| Geometry of the network | S1 | Describe methods used to explore the geometry of the treatment network under study and potential biases related to it. This should include how the evidence base has been graphically summarized for presentation, and what characteristics were compiled and used to describe the evidence base to readers | 8-9 |
| Risk of bias within individual studies | 12 | Describe methods used for assessing the risk of bias of individual studies (including specification of whether this was done at the study or outcome level), and how this information is to be used in any data synthesis. | 8-9 |
| Summary measures | 13 | State the principal summary measures (e.g., risk ratio, difference in means). Also describe the use of additional summary measures assessed, such as treatment rankings and surface under the cumulative ranking curve (SUCRA) values, as well as modified approaches used to present summary findings from meta-analyses. | 8-9 |
| Planned methods of analysis | 14 | Describe the methods of handling data and combining results of studies for each network meta-analysis. This should include, but not be limited to: Handling of multigroup trials; Selection of variance structure; Selection of prior distributions in Bayesian analyses; and Assessment of model fit. | 8-9 |
| Assessment of inconsistency | S2 | Describe the statistical methods used to evaluate the agreement of direct and indirect evidence in the treatment network(s) studied. Describe efforts taken to address its presence when found. | 8-9 |
| Risk of bias across studies | 15 | Specify any assessment of risk of bias that may affect the cumulative evidence (e.g., publication bias, selective reporting within studies) | 8-9 |
| Additional analyses | 16 | Describe methods of additional analyses if done, indicating which were prespecified. This may include, but not be limited to, the following: Sensitivity or subgroup analyses; Meta-regression analyses; Alternative formulations of the treatment network; and Use of alternative prior distributions for Bayesian analyses (if applicable). |  |
| **RESULTS** | | |  |
| Study selection | 17 | Give numbers of studies screened, assessed for eligibility, and included in the review, with reasons for exclusions at each stage, ideally with a flow diagram. | 9-17 |
| Presentation of network structure | S3 | Provide a network graph of the included studies to enable visualization of the geometry of the treatment network. | 9-17 |
| Summary of network geometry | S4 | Provide a brief overview of characteristics of the treatment network. This may include commentary on the abundance of trials and randomized patients for the different interventions and pairwise comparisons in the network, gaps of evidence in the treatment network, and potential biases reflected by the network structure. | 9-17 |
| Study characteristics | 18 | For each study, present characteristics for which data were extracted (e.g., study size, PICOS, follow-up period) and provide the citations. | 9-17 |
| Risk of bias within studies | 19 | Present data on the risk of bias of each study and, if available, any outcome level assessment. | 9-17 |
| Results of individual studies | 20 | For all outcomes considered (benefits or harms), present, for each study: 1) simple summary data for each intervention group, and 2) effect estimates and confidence intervals. Modified approaches may be needed to deal with information from larger networks. | 9-17 |
| Synthesis of results | 21 | Present results of each meta-analysis done, including confidence/credible intervals. In larger networks, authors may focus on comparisons versus a particular comparator (e.g., placebo or standard care), with full findings presented in an appendix. League tables and forest plots may be considered to summarize pairwise comparisons. If additional summary measures were explored (such as treatment rankings), these should also be presented. | 9-17 |
| Exploration for inconsistency | S5 | Describe results from investigations of inconsistency. This may include such information as measures of model fit to compare consistency and inconsistency models, P values from statistical tests, or summary of inconsistency estimates from different parts of the treatment network. | 9-17 |
| Risk of bias across studies | 22 | Present results of any assessment of the risk of bias across studies for the evidence base being studied. | 9-17 |
| Results of additional analyses | 23 | Give results of additional analyses, if done (e.g., sensitivity or subgroup analyses, meta-regression  analyses, alternative network geometries studied, alternative choice of prior distributions for  Bayesian analyses, and so forth). |  |
| **DISCUSSION** | | |  |
| Summary of evidence | 24 | Summarize the main findings, including the strength of evidence for each main outcome; consider their relevance to key groups (e.g., health care providers, researchers, and policymakers). | 17-27 |
| Limitations | 25 | Discuss limitations at study and outcome levels (e.g., risk of bias), and at review level (e.g., incomplete retrieval of identified research, reporting bias). Comment on the validity of the assumptions, such as transitivity and consistency. Comment on any concerns regarding network geometry (e.g., avoidance of certain comparisons). | 25-27 |
| Conclusions | 26 | Provide a general interpretation of the results in the context of other evidence, and implications for future research. | 27 |
| **FUNDING** | | |  |
| Funding | 27 | Describe sources of funding for the systematic review and other support (e.g., supply of data); role of funders for the systematic review. This should also include information regarding whether funding has been received from manufacturers of treatments in the network and/or whether some of the authors are content experts with professional conflicts of interest that could affect the use of treatments in the network. | 28 |

**Appendix 2:** Search Strategies

1 Search Strategies for Web of Science

| NO. | Query |
| --- | --- |
| #1 | stroke (Topic) |
| #2 | TS=('Strokes' OR 'Cerebrovascular Accident*' OR 'Cerebral Stroke*' OR 'Cerebrovascular Apoplexy' OR 'Brain Vascular Accident*' OR 'Cerebrovascular Stroke*' OR 'Apoplexy' OR 'CVA' OR 'CVAs' OR 'Cerebrovascular Accident' OR 'Acute Stroke*' OR 'Acute Cerebrovascular Accident*' OR 'acute cerebrovascular lesion' OR 'acute focal cerebral vasculopathy' OR 'apoplectic stroke' OR 'apoplexia' OR 'brain accident' OR 'brain attack' OR 'brain blood flow disturbance' OR 'brain insult' OR 'brain insultus' OR 'cerebral apoplexia' OR 'cerebral insult' OR 'cerebral vascular accident' OR 'cerebral vascular insufficiency' OR 'cerebro vascular accident' OR 'cerebrovascular arrest' OR 'cerebrovascular failure' OR 'cerebrovascular injury' OR 'cerebrovascular insufficiency' OR 'cerebrovascular insult' OR 'cerebrum vascular accident' OR 'cryptogenic stroke' OR 'insultus cerebralis' OR 'ischaemic seizure' OR 'ischemic seizure' OR 'stroke' OR 'thrombotic stroke') |
| #3 | #1 OR #2 |
| #4 | TS=(exercise) |
| #5 | TS=('Exercises' OR 'Physical Exercise*' OR 'Physical Activity*' OR 'Aerobic Exercise*' OR 'Isometric Exercise*' OR 'Acute Exercise*' OR 'Exercise Training*' OR 'biometric exercise' OR 'effort' OR 'exercise capacity' OR 'exercise performance' OR 'exertion' OR 'fitness training' OR 'fitness workout' OR 'physical effort' OR 'physical exertion' OR 'physical workout' OR 'exercise') |
| #6 | #4 OR #5 |
| #7 | TS=(Resistance Training) |
| #8 | TS=('Strength Training' OR 'Weight Lifting Strengthening Program*' OR 'Weight Lifting Exercise Program*' OR 'Weight Bearing Strengthening Program*' OR 'Weight Bearing Exercise Program*' OR 'resistance exercise' OR 'resistance exercise training' OR 'resistance-type exercise' OR 'resistance-type training' OR 'strength-type exercise' OR 'strength-type training' OR 'resistance training') |
| #9 | #7 OR #8 |
| #10 | TS=(High-Intensity Interval Training) |
| #11 | TS=('High Intensity Interval Training*' OR 'High-Intensity Intermittent Exercise*' OR 'Sprint Interval Training*' OR 'high-intensity intermittent training' OR 'high-intensity interval exercise' OR 'high-intensity interval training' OR 'HIIE' OR 'HIIT' OR 'intermittent high-intensity training' OR 'interval high-intensity training') |
| #12 | #10 OR #11 |
| #13 | #6 OR #9 OR #12 |
| #14 | #3 AND #13 |
| #15 | TS=(Randomized Controlled Trial) |
| #16 | TS=('controlled trial, randomized' OR 'randomised controlled study' OR 'randomised controlled trial' OR 'randomized controlled study' OR 'randomized controlled trial' OR 'rct' OR 'RCT') |
| #17 | #15 OR #16 |
| #18 | #14 AND #17 |

2 Search Strategies for Embase

| No. | Query |
| --- | --- |
| #16 | #14 AND #15 |
| #15 | 'randomized controlled trial'/exp |
| #14 | #3 AND #13 |
| #13 | #6 OR #9 OR #12 |
| #12 | #10 OR #11 |
| #11 | 'high intensity interval training*':ab,ti OR 'high-intensity intermittent exercise*':ab,ti OR 'sprint interval training*':ab,ti OR 'high-intensity intermittent training':ab,ti OR 'high-intensity interval exercise':ab,ti OR 'high-intensity interval training':ab,ti OR 'hiie':ab,ti OR 'hiit':ab,ti OR 'intermittent high-intensity training':ab,ti OR 'interval high-intensity training':ab,ti |
| #10 | 'high intensity interval training'/exp |
| #9 | #7 OR #8 |
| #8 | 'strength training':ab,ti OR 'weight lifting strengthening program*':ab,ti OR 'weight lifting exercise program*':ab,ti OR 'weight bearing strengthening program*':ab,ti OR 'weight bearing exercise program*':ab,ti OR 'resistance exercise':ab,ti OR 'resistance exercise training':ab,ti OR 'resistance-type exercise':ab,ti OR 'resistance-type training':ab,ti OR 'strength-type exercise':ab,ti OR 'strength-type training':ab,ti OR 'resistance training':ab,ti |
| #7 | 'resistance training'/exp |
| #6 | #4 OR #5 |
| #5 | 'exercises':ab,ti OR 'physical exercise*':ab,ti OR 'physical activit*':ab,ti OR 'aerobic exercise*':ab,ti OR 'isometric exercise*':ab,ti OR 'acute exercise*':ab,ti OR 'exercise training*':ab,ti OR 'biometric exercise':ab,ti OR 'effort':ab,ti OR 'exercise capacity':ab,ti OR 'exercise performance':ab,ti OR 'exertion':ab,ti OR 'fitness training':ab,ti OR 'fitness workout':ab,ti OR 'physical effort':ab,ti OR 'physical exertion':ab,ti OR 'physical workout':ab,ti OR 'exercise':ab,ti |
| #4 | 'exercise'/exp |
| #3 | #1 OR #2 |
| #2 | 'strokes':ab,ti OR 'cerebrovascular accident*':ab,ti OR 'cerebral stroke*':ab,ti OR 'cerebrovascular apoplexy':ab,ti OR 'brain vascular accident*':ab,ti OR 'cerebrovascular stroke*':ab,ti OR 'apoplexy':ab,ti OR 'cva':ab,ti OR 'cvas':ab,ti OR 'cerebrovascular accident':ab,ti OR 'acute stroke*':ab,ti OR 'acute cerebrovascular accident*':ab,ti OR 'acute cerebrovascular lesion':ab,ti OR 'acute focal cerebral vasculopathy':ab,ti OR 'apoplectic stroke':ab,ti OR 'apoplexia':ab,ti OR 'brain accident':ab,ti OR 'brain attack':ab,ti OR 'brain blood flow disturbance':ab,ti OR 'brain insult':ab,ti OR 'brain insultus':ab,ti OR 'cerebral apoplexia':ab,ti OR 'cerebral insult':ab,ti OR 'cerebral vascular accident':ab,ti OR 'cerebral vascular insufficiency':ab,ti OR 'cerebro vascular accident':ab,ti OR 'cerebrovascular arrest':ab,ti OR 'cerebrovascular failure':ab,ti OR 'cerebrovascular injury':ab,ti OR 'cerebrovascular insufficiency':ab,ti OR 'cerebrovascular insult':ab,ti OR 'cerebrum vascular accident':ab,ti OR 'cryptogenic stroke':ab,ti OR 'insultus cerebralis':ab,ti OR 'ischaemic seizure':ab,ti OR 'ischemic seizure':ab,ti OR 'stroke':ab,ti OR 'thrombotic stroke':ab,ti |
| #1 | 'cerebrovascular accident'/exp |

3 Search Strategies for PubMed

| NO. | Query |
| --- | --- |
| #1 | "Stroke"[Mesh] |
| #2 | 'Strokes'[Title/Abstract] OR 'Cerebrovascular Accident*'[Title/Abstract] OR 'Cerebral Stroke*'[Title/Abstract] OR 'Cerebrovascular Apoplexy'[Title/Abstract] OR 'Brain Vascular Accident*'[Title/Abstract] OR 'Cerebrovascular Stroke*'[Title/Abstract] OR 'Apoplexy'[Title/Abstract] OR 'CVA'[Title/Abstract] OR 'CVAs'[Title/Abstract] OR 'Cerebrovascular Accident'[Title/Abstract] OR 'Acute Stroke*'[Title/Abstract] OR 'Acute Cerebrovascular Accident*'[Title/Abstract] OR 'acute cerebrovascular lesion'[Title/Abstract] OR 'acute focal cerebral vasculopathy'[Title/Abstract] OR 'apoplectic stroke'[Title/Abstract] OR 'apoplexia'[Title/Abstract] OR 'brain accident'[Title/Abstract] OR 'brain attack'[Title/Abstract] OR 'brain blood flow disturbance'[Title/Abstract] OR 'brain insult'[Title/Abstract] OR 'brain insultus'[Title/Abstract] OR 'cerebral apoplexia'[Title/Abstract] OR 'cerebral insult'[Title/Abstract] OR 'cerebral vascular accident'[Title/Abstract] OR 'cerebral vascular insufficiency'[Title/Abstract] OR 'cerebro vascular accident'[Title/Abstract] OR 'cerebrovascular arrest'[Title/Abstract] OR 'cerebrovascular failure'[Title/Abstract] OR 'cerebrovascular injury'[Title/Abstract] OR 'cerebrovascular insufficiency'[Title/Abstract] OR 'cerebrovascular insult'[Title/Abstract] OR 'cerebrum vascular accident'[Title/Abstract] OR 'cryptogenic stroke'[Title/Abstract] OR 'insultus cerebralis'[Title/Abstract] OR 'ischaemic seizure'[Title/Abstract] OR 'ischemic seizure'[Title/Abstract] OR 'stroke'[Title/Abstract] OR 'thrombotic stroke'[Title/Abstract] |
| #3 | ("Stroke"[Mesh]) OR ('Strokes'[Title/Abstract] OR 'Cerebrovascular Accident*'[Title/Abstract] OR 'Cerebral Stroke*'[Title/Abstract] OR 'Cerebrovascular Apoplexy'[Title/Abstract] OR 'Brain Vascular Accident*'[Title/Abstract] OR 'Cerebrovascular Stroke*'[Title/Abstract] OR 'Apoplexy'[Title/Abstract] OR 'CVA'[Title/Abstract] OR 'CVAs'[Title/Abstract] OR 'Cerebrovascular Accident'[Title/Abstract] OR 'Acute Stroke*'[Title/Abstract] OR 'Acute Cerebrovascular Accident*'[Title/Abstract] OR 'acute cerebrovascular lesion'[Title/Abstract] OR 'acute focal cerebral vasculopathy'[Title/Abstract] OR 'apoplectic stroke'[Title/Abstract] OR 'apoplexia'[Title/Abstract] OR 'brain accident'[Title/Abstract] OR 'brain attack'[Title/Abstract] OR 'brain blood flow disturbance'[Title/Abstract] OR 'brain insult'[Title/Abstract] OR 'brain insultus'[Title/Abstract] OR 'cerebral apoplexia'[Title/Abstract] OR 'cerebral insult'[Title/Abstract] OR 'cerebral vascular accident'[Title/Abstract] OR 'cerebral vascular insufficiency'[Title/Abstract] OR 'cerebro vascular accident'[Title/Abstract] OR 'cerebrovascular arrest'[Title/Abstract] OR 'cerebrovascular failure'[Title/Abstract] OR 'cerebrovascular injury'[Title/Abstract] OR 'cerebrovascular insufficiency'[Title/Abstract] OR 'cerebrovascular insult'[Title/Abstract] OR 'cerebrum vascular accident'[Title/Abstract] OR 'cryptogenic stroke'[Title/Abstract] OR 'insultus cerebralis'[Title/Abstract] OR 'ischaemic seizure'[Title/Abstract] OR 'ischemic seizure'[Title/Abstract] OR 'stroke'[Title/Abstract] OR 'thrombotic stroke'[Title/Abstract]) |
| #4 | "Exercise"[Mesh] |
| #5 | 'Exercises'[Title/Abstract] OR 'Physical Exercise*'[Title/Abstract] OR 'Physical Activit*'[Title/Abstract] OR 'Aerobic Exercise*'[Title/Abstract] OR 'Isometric Exercise*'[Title/Abstract] OR 'Acute Exercise*'[Title/Abstract] OR 'Exercise Training*'[Title/Abstract] OR 'biometric exercise'[Title/Abstract] OR 'effort'[Title/Abstract] OR 'exercise capacity'[Title/Abstract] OR 'exercise performance'[Title/Abstract] OR 'exertion'[Title/Abstract] OR 'fitness training'[Title/Abstract] OR 'fitness workout'[Title/Abstract] OR 'physical effort'[Title/Abstract] OR 'physical exertion'[Title/Abstract] OR 'physical workout'[Title/Abstract] OR 'exercise'[Title/Abstract] |
| #6 | ("Exercise"[Mesh]) OR ('Exercises'[Title/Abstract] OR 'Physical Exercise*'[Title/Abstract] OR 'Physical Activit*'[Title/Abstract] OR 'Aerobic Exercise*'[Title/Abstract] OR 'Isometric Exercise*'[Title/Abstract] OR 'Acute Exercise*'[Title/Abstract] OR 'Exercise Training*'[Title/Abstract] OR 'biometric exercise'[Title/Abstract] OR 'effort'[Title/Abstract] OR 'exercise capacity'[Title/Abstract] OR 'exercise performance'[Title/Abstract] OR 'exertion'[Title/Abstract] OR 'fitness training'[Title/Abstract] OR 'fitness workout'[Title/Abstract] OR 'physical effort'[Title/Abstract] OR 'physical exertion'[Title/Abstract] OR 'physical workout'[Title/Abstract] OR 'exercise'[Title/Abstract]) |
| #7 | "Resistance Training"[Mesh] |
| #8 | 'Strength Training'[Title/Abstract] OR 'Weight Lifting Strengthening Program*'[Title/Abstract] OR 'Weight Lifting Exercise Program*'[Title/Abstract] OR 'Weight Bearing Strengthening Program*'[Title/Abstract] OR 'Weight Bearing Exercise Program*'[Title/Abstract] OR 'resistance exercise'[Title/Abstract] OR 'resistance exercise training'[Title/Abstract] OR 'resistance-type exercise'[Title/Abstract] OR 'resistance-type training'[Title/Abstract] OR 'strength-type exercise'[Title/Abstract] OR 'strength-type training'[Title/Abstract] OR 'resistance training'[Title/Abstract] |
| #9 | ("Resistance Training"[Mesh]) OR ('Strength Training'[Title/Abstract] OR 'Weight Lifting Strengthening Program*'[Title/Abstract] OR 'Weight Lifting Exercise Program*'[Title/Abstract] OR 'Weight Bearing Strengthening Program*'[Title/Abstract] OR 'Weight Bearing Exercise Program*'[Title/Abstract] OR 'resistance exercise'[Title/Abstract] OR 'resistance exercise training'[Title/Abstract] OR 'resistance-type exercise'[Title/Abstract] OR 'resistance-type training'[Title/Abstract] OR 'strength-type exercise'[Title/Abstract] OR 'strength-type training'[Title/Abstract] OR 'resistance training'[Title/Abstract]) |
| #10 | "High-Intensity Interval Training"[Mesh] |
| #11 | 'High Intensity Interval Training*'[Title/Abstract] OR 'High-Intensity Intermittent Exercise*'[Title/Abstract] OR 'Sprint Interval Training*'[Title/Abstract] OR 'high-intensity intermittent training'[Title/Abstract] OR 'high-intensity interval exercise'[Title/Abstract] OR 'high-intensity interval training'[Title/Abstract] OR 'HIIE'[Title/Abstract] OR 'HIIT'[Title/Abstract] OR 'intermittent high-intensity training'[Title/Abstract] OR 'interval high-intensity training'[Title/Abstract] |
| #12 | ("High-Intensity Interval Training"[Mesh]) OR ('High Intensity Interval Training*'[Title/Abstract] OR 'High-Intensity Intermittent Exercise*'[Title/Abstract] OR 'Sprint Interval Training*'[Title/Abstract] OR 'high-intensity intermittent training'[Title/Abstract] OR 'high-intensity interval exercise'[Title/Abstract] OR 'high-intensity interval training'[Title/Abstract] OR 'HIIE'[Title/Abstract] OR 'HIIT'[Title/Abstract] OR 'intermittent high-intensity training'[Title/Abstract] OR 'interval high-intensity training'[Title/Abstract]) |
| #13 | ((("Exercise"[Mesh]) OR ('Exercises'[Title/Abstract] OR 'Physical Exercise*'[Title/Abstract] OR 'Physical Activit*'[Title/Abstract] OR 'Aerobic Exercise*'[Title/Abstract] OR 'Isometric Exercise*'[Title/Abstract] OR 'Acute Exercise*'[Title/Abstract] OR 'Exercise Training*'[Title/Abstract] OR 'biometric exercise'[Title/Abstract] OR 'effort'[Title/Abstract] OR 'exercise capacity'[Title/Abstract] OR 'exercise performance'[Title/Abstract] OR 'exertion'[Title/Abstract] OR 'fitness training'[Title/Abstract] OR 'fitness workout'[Title/Abstract] OR 'physical effort'[Title/Abstract] OR 'physical exertion'[Title/Abstract] OR 'physical workout'[Title/Abstract] OR 'exercise'[Title/Abstract])) OR (("Resistance Training"[Mesh]) OR ('Strength Training'[Title/Abstract] OR 'Weight Lifting Strengthening Program*'[Title/Abstract] OR 'Weight Lifting Exercise Program*'[Title/Abstract] OR 'Weight Bearing Strengthening Program*'[Title/Abstract] OR 'Weight Bearing Exercise Program*'[Title/Abstract] OR 'resistance exercise'[Title/Abstract] OR 'resistance exercise training'[Title/Abstract] OR 'resistance-type exercise'[Title/Abstract] OR 'resistance-type training'[Title/Abstract] OR 'strength-type exercise'[Title/Abstract] OR 'strength-type training'[Title/Abstract] OR 'resistance training'[Title/Abstract]))) OR (("High-Intensity Interval Training"[Mesh]) OR ('High Intensity Interval Training*'[Title/Abstract] OR 'High-Intensity Intermittent Exercise*'[Title/Abstract] OR 'Sprint Interval Training*'[Title/Abstract] OR 'high-intensity intermittent training'[Title/Abstract] OR 'high-intensity interval exercise'[Title/Abstract] OR 'high-intensity interval training'[Title/Abstract] OR 'HIIE'[Title/Abstract] OR 'HIIT'[Title/Abstract] OR 'intermittent high-intensity training'[Title/Abstract] OR 'interval high-intensity training'[Title/Abstract])) |
| #14 | (("Stroke"[Mesh]) OR ('Strokes'[Title/Abstract] OR 'Cerebrovascular Accident*'[Title/Abstract] OR 'Cerebral Stroke*'[Title/Abstract] OR 'Cerebrovascular Apoplexy'[Title/Abstract] OR 'Brain Vascular Accident*'[Title/Abstract] OR 'Cerebrovascular Stroke*'[Title/Abstract] OR 'Apoplexy'[Title/Abstract] OR 'CVA'[Title/Abstract] OR 'CVAs'[Title/Abstract] OR 'Cerebrovascular Accident'[Title/Abstract] OR 'Acute Stroke*'[Title/Abstract] OR 'Acute Cerebrovascular Accident*'[Title/Abstract] OR 'acute cerebrovascular lesion'[Title/Abstract] OR 'acute focal cerebral vasculopathy'[Title/Abstract] OR 'apoplectic stroke'[Title/Abstract] OR 'apoplexia'[Title/Abstract] OR 'brain accident'[Title/Abstract] OR 'brain attack'[Title/Abstract] OR 'brain blood flow disturbance'[Title/Abstract] OR 'brain insult'[Title/Abstract] OR 'brain insultus'[Title/Abstract] OR 'cerebral apoplexia'[Title/Abstract] OR 'cerebral insult'[Title/Abstract] OR 'cerebral vascular accident'[Title/Abstract] OR 'cerebral vascular insufficiency'[Title/Abstract] OR 'cerebro vascular accident'[Title/Abstract] OR 'cerebrovascular arrest'[Title/Abstract] OR 'cerebrovascular failure'[Title/Abstract] OR 'cerebrovascular injury'[Title/Abstract] OR 'cerebrovascular insufficiency'[Title/Abstract] OR 'cerebrovascular insult'[Title/Abstract] OR 'cerebrum vascular accident'[Title/Abstract] OR 'cryptogenic stroke'[Title/Abstract] OR 'insultus cerebralis'[Title/Abstract] OR 'ischaemic seizure'[Title/Abstract] OR 'ischemic seizure'[Title/Abstract] OR 'stroke'[Title/Abstract] OR 'thrombotic stroke'[Title/Abstract])) AND (((("Exercise"[Mesh]) OR ('Exercises'[Title/Abstract] OR 'Physical Exercise*'[Title/Abstract] OR 'Physical Activit*'[Title/Abstract] OR 'Aerobic Exercise*'[Title/Abstract] OR 'Isometric Exercise*'[Title/Abstract] OR 'Acute Exercise*'[Title/Abstract] OR 'Exercise Training*'[Title/Abstract] OR 'biometric exercise'[Title/Abstract] OR 'effort'[Title/Abstract] OR 'exercise capacity'[Title/Abstract] OR 'exercise performance'[Title/Abstract] OR 'exertion'[Title/Abstract] OR 'fitness training'[Title/Abstract] OR 'fitness workout'[Title/Abstract] OR 'physical effort'[Title/Abstract] OR 'physical exertion'[Title/Abstract] OR 'physical workout'[Title/Abstract] OR 'exercise'[Title/Abstract])) OR (("Resistance Training"[Mesh]) OR ('Strength Training'[Title/Abstract] OR 'Weight Lifting Strengthening Program*'[Title/Abstract] OR 'Weight Lifting Exercise Program*'[Title/Abstract] OR 'Weight Bearing Strengthening Program*'[Title/Abstract] OR 'Weight Bearing Exercise Program*'[Title/Abstract] OR 'resistance exercise'[Title/Abstract] OR 'resistance exercise training'[Title/Abstract] OR 'resistance-type exercise'[Title/Abstract] OR 'resistance-type training'[Title/Abstract] OR 'strength-type exercise'[Title/Abstract] OR 'strength-type training'[Title/Abstract] OR 'resistance training'[Title/Abstract]))) OR (("High-Intensity Interval Training"[Mesh]) OR ('High Intensity Interval Training*'[Title/Abstract] OR 'High-Intensity Intermittent Exercise*'[Title/Abstract] OR 'Sprint Interval Training*'[Title/Abstract] OR 'high-intensity intermittent training'[Title/Abstract] OR 'high-intensity interval exercise'[Title/Abstract] OR 'high-intensity interval training'[Title/Abstract] OR 'HIIE'[Title/Abstract] OR 'HIIT'[Title/Abstract] OR 'intermittent high-intensity training'[Title/Abstract] OR 'interval high-intensity training'[Title/Abstract]))) |
| #15 | "Randomized Controlled Trial" [Publication Type] |
| #16 | 'controlled trial, randomized'[Title/Abstract] OR 'randomised controlled study'[Title/Abstract] OR 'randomised controlled trial'[Title/Abstract] OR 'randomized controlled study'[Title/Abstract] OR 'randomized controlled trial'[Title/Abstract] OR 'rct'[Title/Abstract] OR 'RCT'[Title/Abstract] |
| #17 | ("Randomized Controlled Trial" [Publication Type]) OR ('controlled trial, randomized'[Title/Abstract] OR 'randomised controlled study'[Title/Abstract] OR 'randomised controlled trial'[Title/Abstract] OR 'randomized controlled study'[Title/Abstract] OR 'randomized controlled trial'[Title/Abstract] OR 'rct'[Title/Abstract] OR 'RCT'[Title/Abstract]) |
| #18 | ((("Stroke"[Mesh]) OR ('Strokes'[Title/Abstract] OR 'Cerebrovascular Accident*'[Title/Abstract] OR 'Cerebral Stroke*'[Title/Abstract] OR 'Cerebrovascular Apoplexy'[Title/Abstract] OR 'Brain Vascular Accident*'[Title/Abstract] OR 'Cerebrovascular Stroke*'[Title/Abstract] OR 'Apoplexy'[Title/Abstract] OR 'CVA'[Title/Abstract] OR 'CVAs'[Title/Abstract] OR 'Cerebrovascular Accident'[Title/Abstract] OR 'Acute Stroke*'[Title/Abstract] OR 'Acute Cerebrovascular Accident*'[Title/Abstract] OR 'acute cerebrovascular lesion'[Title/Abstract] OR 'acute focal cerebral vasculopathy'[Title/Abstract] OR 'apoplectic stroke'[Title/Abstract] OR 'apoplexia'[Title/Abstract] OR 'brain accident'[Title/Abstract] OR 'brain attack'[Title/Abstract] OR 'brain blood flow disturbance'[Title/Abstract] OR 'brain insult'[Title/Abstract] OR 'brain insultus'[Title/Abstract] OR 'cerebral apoplexia'[Title/Abstract] OR 'cerebral insult'[Title/Abstract] OR 'cerebral vascular accident'[Title/Abstract] OR 'cerebral vascular insufficiency'[Title/Abstract] OR 'cerebro vascular accident'[Title/Abstract] OR 'cerebrovascular arrest'[Title/Abstract] OR 'cerebrovascular failure'[Title/Abstract] OR 'cerebrovascular injury'[Title/Abstract] OR 'cerebrovascular insufficiency'[Title/Abstract] OR 'cerebrovascular insult'[Title/Abstract] OR 'cerebrum vascular accident'[Title/Abstract] OR 'cryptogenic stroke'[Title/Abstract] OR 'insultus cerebralis'[Title/Abstract] OR 'ischaemic seizure'[Title/Abstract] OR 'ischemic seizure'[Title/Abstract] OR 'stroke'[Title/Abstract] OR 'thrombotic stroke'[Title/Abstract])) AND (((("Exercise"[Mesh]) OR ('Exercises'[Title/Abstract] OR 'Physical Exercise*'[Title/Abstract] OR 'Physical Activit*'[Title/Abstract] OR 'Aerobic Exercise*'[Title/Abstract] OR 'Isometric Exercise*'[Title/Abstract] OR 'Acute Exercise*'[Title/Abstract] OR 'Exercise Training*'[Title/Abstract] OR 'biometric exercise'[Title/Abstract] OR 'effort'[Title/Abstract] OR 'exercise capacity'[Title/Abstract] OR 'exercise performance'[Title/Abstract] OR 'exertion'[Title/Abstract] OR 'fitness training'[Title/Abstract] OR 'fitness workout'[Title/Abstract] OR 'physical effort'[Title/Abstract] OR 'physical exertion'[Title/Abstract] OR 'physical workout'[Title/Abstract] OR 'exercise'[Title/Abstract])) OR (("Resistance Training"[Mesh]) OR ('Strength Training'[Title/Abstract] OR 'Weight Lifting Strengthening Program*'[Title/Abstract] OR 'Weight Lifting Exercise Program*'[Title/Abstract] OR 'Weight Bearing Strengthening Program*'[Title/Abstract] OR 'Weight Bearing Exercise Program*'[Title/Abstract] OR 'resistance exercise'[Title/Abstract] OR 'resistance exercise training'[Title/Abstract] OR 'resistance-type exercise'[Title/Abstract] OR 'resistance-type training'[Title/Abstract] OR 'strength-type exercise'[Title/Abstract] OR 'strength-type training'[Title/Abstract] OR 'resistance training'[Title/Abstract]))) OR (("High-Intensity Interval Training"[Mesh]) OR ('High Intensity Interval Training*'[Title/Abstract] OR 'High-Intensity Intermittent Exercise*'[Title/Abstract] OR 'Sprint Interval Training*'[Title/Abstract] OR 'high-intensity intermittent training'[Title/Abstract] OR 'high-intensity interval exercise'[Title/Abstract] OR 'high-intensity interval training'[Title/Abstract] OR 'HIIE'[Title/Abstract] OR 'HIIT'[Title/Abstract] OR 'intermittent high-intensity training'[Title/Abstract] OR 'interval high-intensity training'[Title/Abstract])))) AND (("Randomized Controlled Trial" [Publication Type]) OR ('controlled trial, randomized'[Title/Abstract] OR 'randomised controlled study'[Title/Abstract] OR 'randomised controlled trial'[Title/Abstract] OR 'randomized controlled study'[Title/Abstract] OR 'randomized controlled trial'[Title/Abstract] OR 'rct'[Title/Abstract] OR 'RCT'[Title/Abstract])) |

4 Search Strategies for Cochrane Library

| NO. | Query |
| --- | --- |
| #1 | MeSH descriptor: [Stroke] explode all trees |
| #2 | ('Strokes' OR 'Cerebrovascular Accident*' OR 'Cerebral Stroke*' OR 'Cerebrovascular Apoplexy' OR 'Brain Vascular Accident*' OR 'Cerebrovascular Stroke*' OR 'Apoplexy' OR 'CVA' OR 'CVAs' OR 'Cerebrovascular Accident' OR 'Acute Stroke*' OR 'Acute Cerebrovascular Accident*' OR 'acute cerebrovascular lesion' OR 'acute focal cerebral vasculopathy' OR 'apoplectic stroke' OR 'apoplexia' OR 'brain accident' OR 'brain attack' OR 'brain blood flow disturbance' OR 'brain insult' OR 'brain insultus' OR 'cerebral apoplexia' OR 'cerebral insult' OR 'cerebral vascular accident' OR 'cerebral vascular insufficiency' OR 'cerebro vascular accident' OR 'cerebrovascular arrest' OR 'cerebrovascular failure' OR 'cerebrovascular injury' OR 'cerebrovascular insufficiency' OR 'cerebrovascular insult' OR 'cerebrum vascular accident' OR 'cryptogenic stroke' OR 'insultus cerebralis' OR 'ischaemic seizure' OR 'ischemic seizure' OR 'stroke' OR 'thrombotic stroke'):ti,ab,kw (Word variations have been searched) |
| #3 | #1 OR #2 |
| #4 | MeSH descriptor: [Exercise] explode all trees |
| #5 | ('Exercises' OR 'Physical Exercise*' OR 'Physical Activity*' OR 'Aerobic Exercise*' OR 'Isometric Exercise*' OR 'Acute Exercise*' OR 'Exercise Training*' OR 'biometric exercise' OR 'effort' OR 'exercise capacity' OR 'exercise performance' OR 'exertion' OR 'fitness training' OR 'fitness workout' OR 'physical effort' OR 'physical exertion' OR 'physical workout' OR 'exercise'):ti,ab,kw (Word variations have been searched) |
| #6 | #4 OR #5 |
| #7 | MeSH descriptor: [Resistance Training] explode all trees |
| #8 | ('Strength Training' OR 'Weight Lifting Strengthening Program*' OR 'Weight Lifting Exercise Program*' OR 'Weight Bearing Strengthening Program*' OR 'Weight Bearing Exercise Program*' OR 'resistance exercise' OR 'resistance exercise training' OR 'resistance-type exercise' OR 'resistance-type training' OR 'strength-type exercise' OR 'strength-type training' OR 'resistance training'):ti,ab,kw (Word variations have been searched) |
| #9 | #7 OR #8 |
| #10 | MeSH descriptor: [High-Intensity Interval Training] explode all trees |
| #11 | ('High Intensity Interval Training*' OR 'High-Intensity Intermittent Exercise*' OR 'Sprint Interval Training*' OR 'high-intensity intermittent training' OR 'high-intensity interval exercise' OR 'high-intensity interval training' OR 'HIIE' OR 'HIIT' OR 'intermittent high-intensity training' OR 'interval high-intensity training'):ti,ab,kw (Word variations have been searched) |
| #12 | #10 OR #11 |
| #13 | #6 OR #9 OR #12 |
| #14 | #3 AND #13 |
| #15 | MeSH descriptor: [Randomized Controlled Trial] explode all trees |
| #16 | ('controlled trial, randomized' OR 'randomised controlled study' OR 'randomised controlled trial' OR 'randomized controlled study' OR 'randomized controlled trial' OR 'rct' OR 'RCT'):ti,ab,kw (Word variations have been searched) |
| #17 | #15 OR #16 |
| #18 | #14 AND #17 |

**Table S1:** GRADE Evidence Results of the BBS Indicator

| **Comparison** | **Within-study bias** | **Reporting bias** | **Indirectness** | **Imprecision** | **Heterogeneity** | **Incoherence** | **Confidence rating** | **Reason(s) for downgrading** |
| --- | --- | --- | --- | --- | --- | --- | --- | --- |
| AE:CT | Some concerns | Low risk | Some concerns | Some concerns | Major concerns | No concerns | Very low | ["Imprecision","Heterogeneity"] |
| AE:HIIT | Some concerns | Low risk | Some concerns | Major concerns | No concerns | No concerns | Low | ["Imprecision"] |
| AE:Mixed | Major concerns | Low risk | Some concerns | Major concerns | Major concerns | No concerns | Very low | ["Within-study bias","Imprecision ","Heterogeneity"] |
| CSE:CT | Some concerns | Low risk | Some concerns | Some concerns | Major concerns | No concerns | Very low | ["Imprecision","Heterogeneity"] |
| CSE:RT | Some concerns | Low risk | Some concerns | Major concerns | Major concerns | No concerns | Very low | ["Imprecision","Heterogeneity"] |
| CT:Mixed | Major concerns | Low risk | Some concerns | No concerns | Major concerns | No concerns | Very low | ["Within-study bias","Heterogeneity"] |
| CT:PME | Some concerns | Low risk | Some concerns | Major concerns | Major concerns | No concerns | Very low | ["Imprecision","Heterogeneity"] |
| CT:RT | Major concerns | Low risk | Some concerns | Some concerns | Major concerns | No concerns | Very low | ["Within-study bias","Imprecision ","Heterogeneity"] |
| AE:CSE | Some concerns | Low risk | Some concerns | Major concerns | No concerns | Some concerns | Very low | ["Imprecision","Incoherence"] |
| AE:PME | Some concerns | Low risk | Some concerns | Major concerns | No concerns | Some concerns | Very low | ["Imprecision","Incoherence"] |
| AE:RT | Major concerns | Low risk | Some concerns | Major concerns | No concerns | Some concerns | Very low | ["Within-study bias","Imprecision","Incoherence"] |
| CSE:HIIT | Some concerns | Low risk | Some concerns | Major concerns | No concerns | Some concerns | Very low | ["Imprecision","Incoherence"] |
| CSE:Mixed | Major concerns | Low risk | Some concerns | Major concerns | No concerns | Some concerns | Very low | ["Within-study bias","Imprecision","Incoherence"] |
| CSE:PME | Some concerns | Low risk | Some concerns | Major concerns | No concerns | Some concerns | Very low | ["Imprecision","Incoherence"] |
| CT:HIIT | Some concerns | Low risk | Some concerns | Major concerns | No concerns | Some concerns | Very low | ["Imprecision","Incoherence"] |
| HIIT:Mixed | Major concerns | Low risk | Some concerns | Major concerns | No concerns | Some concerns | Very low | ["Within-study bias","Imprecision","Incoherence"] |
| HIIT:PME | Some concerns | Low risk | Some concerns | Major concerns | No concerns | Some concerns | Very low | ["Imprecision","Incoherence"] |
| HIIT:RT | Some concerns | Low risk | Some concerns | Major concerns | No concerns | Some concerns | Very low | ["Imprecision","Incoherence"] |
| Mixed:PME | Some concerns | Low risk | Some concerns | Major concerns | No concerns | Some concerns | Very low | ["Imprecision","Incoherence"] |
| Mixed:RT | Major concerns | Low risk | Some concerns | Major concerns | No concerns | Some concerns | Very low | ["Within-study bias","Imprecision","Incoherence"] |
| PME:RT | Some concerns | Low risk | Some concerns | Major concerns | No concerns | Some concerns | Very low | ["Imprecision","Incoherence"] |

**Table S2:** GRADE Evidence Results of the TUG Indicator

| **Comparison** | **Within-study bias** | **Reporting bias** | **Indirectness** | **Imprecision** | **Heterogeneity** | **Incoherence** | **Confidence rating** | **Reason(s) for downgrading** |
| --- | --- | --- | --- | --- | --- | --- | --- | --- |
| AE:CT | Some concerns | Low risk | Some concerns | No concerns | No concerns | No concerns | Moderate | ["Within-study bias","Indirectness"] |
| CSE:CT | Some concerns | Low risk | Some concerns | Major concerns | No concerns | No concerns | Low | ["Imprecision"] |
| CSE:RT | Some concerns | Low risk | Some concerns | Major concerns | Major concerns | No concerns | Very low | ["Imprecision","Heterogeneity"] |
| CT:Mixed | Major concerns | Low risk | Some concerns | Major concerns | No concerns | No concerns | Very low | ["Within-study bias","Imprecision"] |
| CT:PME | Some concerns | Low risk | Some concerns | Major concerns | Major concerns | No concerns | Very low | ["Imprecision","Heterogeneity"] |
| CT:RT | Major concerns | Low risk | Some concerns | Major concerns | Major concerns | No concerns | Very low | ["Within-study bias","Imprecision","Heterogeneity"] |
| PME:RT | Some concerns | Low risk | Some concerns | Major concerns | Major concerns | No concerns | Very low | ["Imprecision","Heterogeneity"] |
| AE:CSE | Some concerns | Low risk | Some concerns | Major concerns | No concerns | Some concerns | Very low | ["Imprecision","Incoherence"] |
| AE:Mixed | Major concerns | Low risk | Some concerns | Major concerns | No concerns | Some concerns | Very low | ["Within-study bias","Imprecision","Incoherence"] |
| AE:PME | Some concerns | Low risk | Some concerns | Major concerns | No concerns | Some concerns | Very low | ["Imprecision","Incoherence"] |
| AE:RT | Some concerns | Low risk | Some concerns | Major concerns | No concerns | Some concerns | Very low | ["Imprecision","Incoherence"] |
| CSE:Mixed | Major concerns | Low risk | Some concerns | Major concerns | No concerns | Some concerns | Very low | ["Within-study bias","Imprecision","Incoherence"] |
| CSE:PME | Some concerns | Low risk | Some concerns | Major concerns | No concerns | Some concerns | Very low | ["Imprecision","Incoherence"] |
| Mixed:PME | Major concerns | Low risk | Some concerns | Major concerns | No concerns | Some concerns | Very low | ["Within-study bias","Imprecision","Incoherence"] |
| Mixed:RT | Major concerns | Low risk | Some concerns | Major concerns | No concerns | Some concerns | Very low | ["Within-study bias","Imprecision","Incoherence"] |

**Table S3:** GRADE Evidence Results of the MoCA Indicator

| **Comparison** | **Within-study bias** | **Reporting bias** | **Indirectness** | **Imprecision** | **Heterogeneity** | **Incoherence** | **Confidence rating** | **Reason(s) for downgrading** |
| --- | --- | --- | --- | --- | --- | --- | --- | --- |
| AE:CT | Major concerns | Low risk | Some concerns | No concerns | No concerns | No concerns | Moderate | ["Within-study bias"] |
| AE:Mixed | Major concerns | Low risk | Some concerns | No concerns | No concerns | No concerns | Moderate | ["Within-study bias"] |
| CT:HIIT | Major concerns | Low risk | Some concerns | Some concerns | No concerns | No concerns | Low | ["Within-study bias","Imprecision"] |
| CT:Mixed | Major concerns | Low risk | Some concerns | Some concerns | No concerns | No concerns | Low | ["Within-study bias","Imprecision"] |
| CT:PME | Major concerns | Low risk | Some concerns | No concerns | No concerns | No concerns | Moderate | ["Within-study bias"] |
| AE:HIIT | Major concerns | Low risk | Some concerns | Major concerns | No concerns | Some concerns | Very low | ["Within-study bias","Imprecision","Incoherence"] |
| AE:PME | Major concerns | Low risk | Some concerns | Major concerns | No concerns | Some concerns | Very low | ["Within-study bias","Imprecision","Incoherence"] |
| HIIT:Mixed | Major concerns | Low risk | Some concerns | Major concerns | No concerns | Some concerns | Very low | ["Within-study bias","Imprecision","Incoherence"] |
| HIIT:PME | Major concerns | Low risk | Some concerns | Some concerns | No concerns | Some concerns | Very low | ["Within-study bias","Imprecision","Incoherence"] |
| Mixed:PME | Major concerns | Low risk | Some concerns | No concerns | No concerns | Some concerns | Very low | ["Within-study bias","Incoherence"] |

**
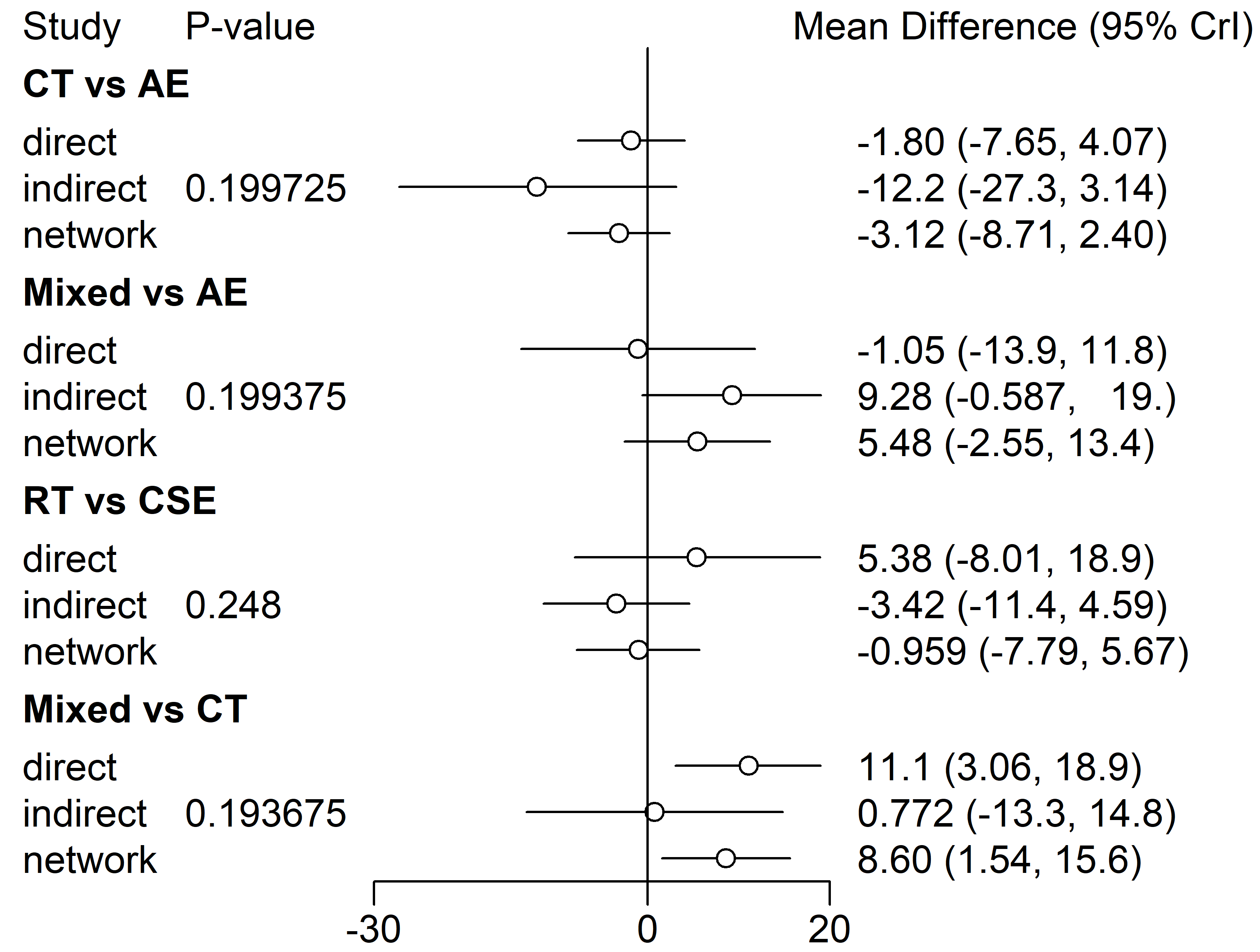
**

**Figure S1:** Node-splitting method results for the BBS indicato


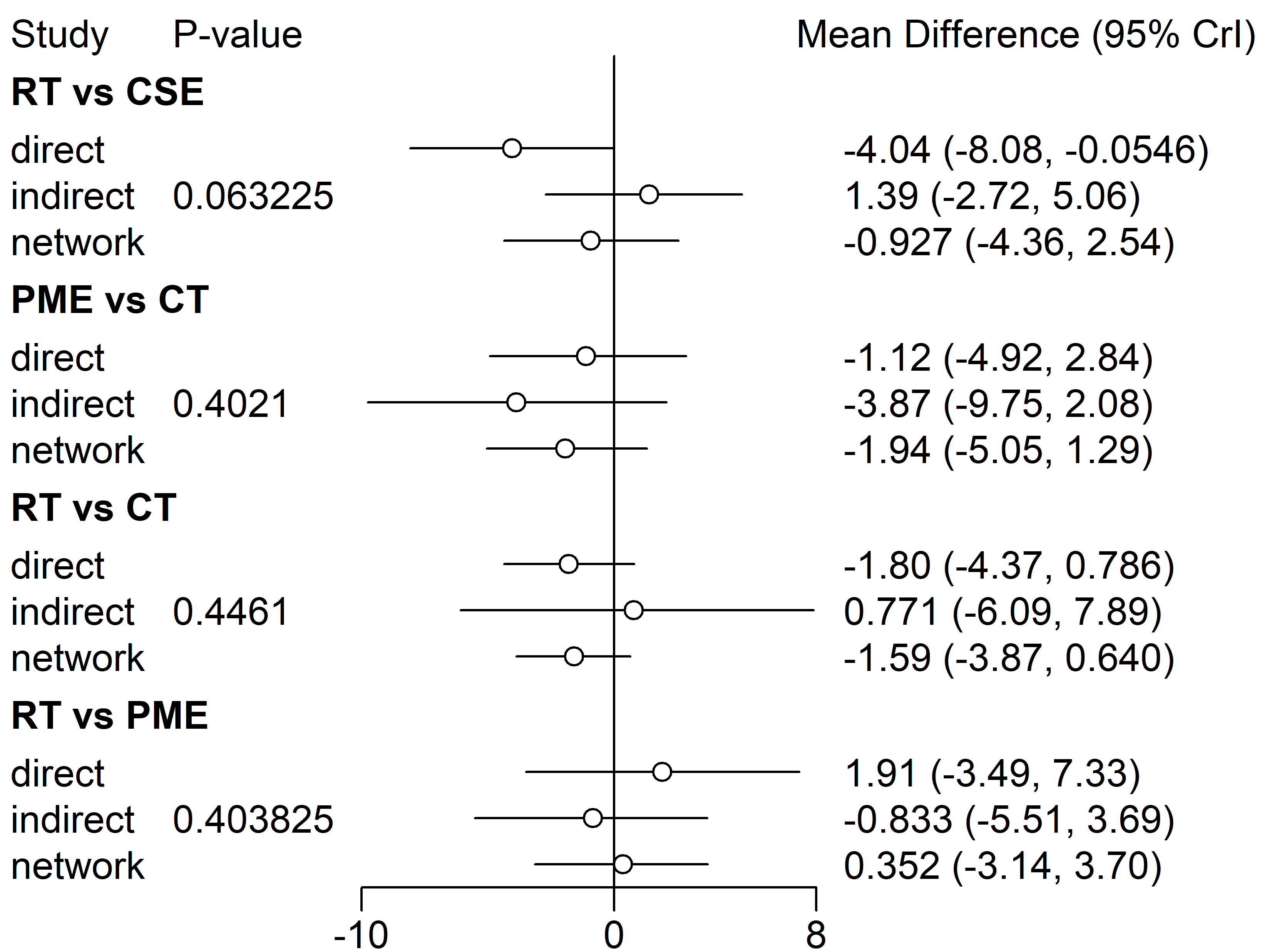


**Figure S2:** Node-splitting method results for the TUG indicato


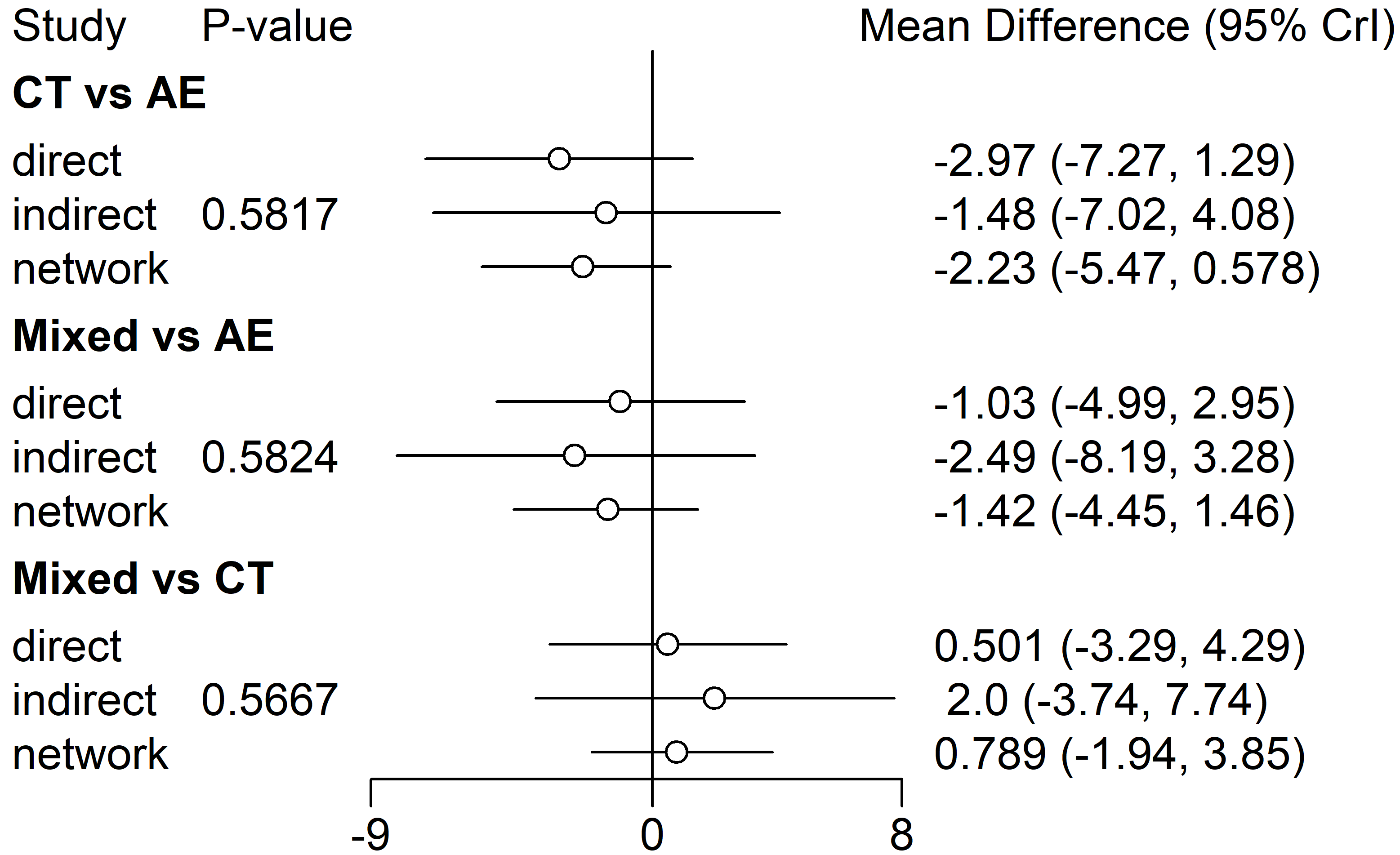


**Figure S3:** Node-splitting method results for the MoCA indicato


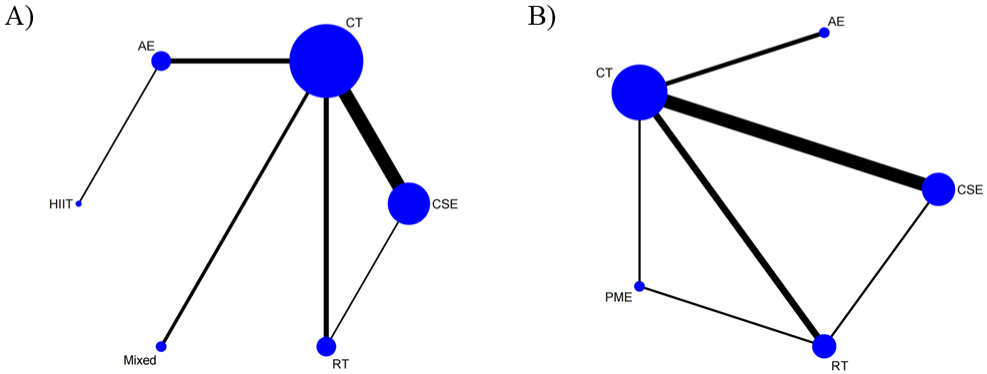


**Figure S4:** Network plot of the effectiveness of each intervention (duration <12 weeks) on BBS, TUG, and MoCA Scores. A) BBS; B) TUG. The points in the figure represent various interventions, with the size of each point indicating the sample size. The lines connecting two points illustrate direct comparisons between different interventions, where a thicker line signifies a greater number of corresponding studies.


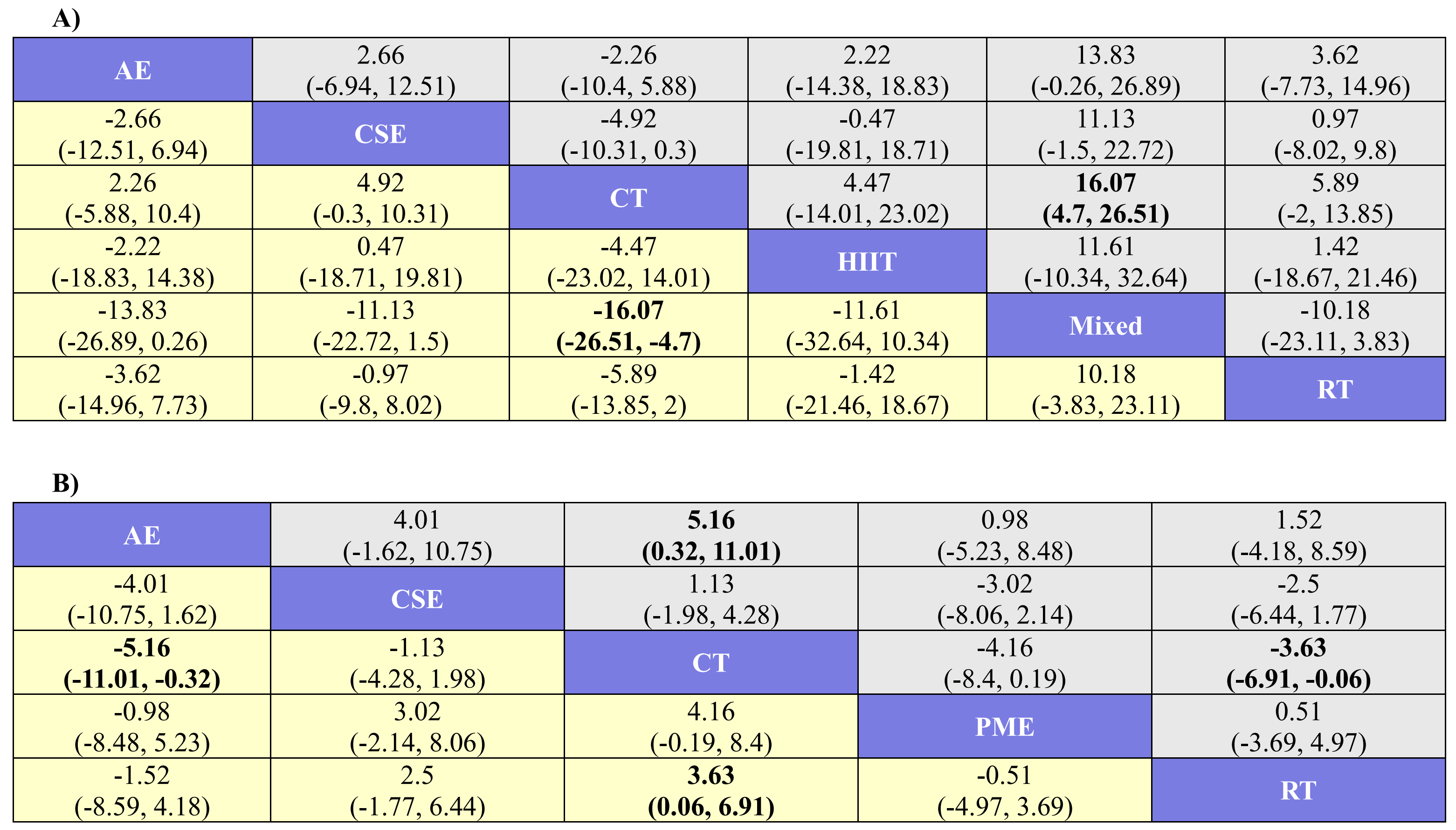


**Figure S5:** League tables for each outcome indicator in the <12-week intervention subgroup A) BBS; B) TUG.


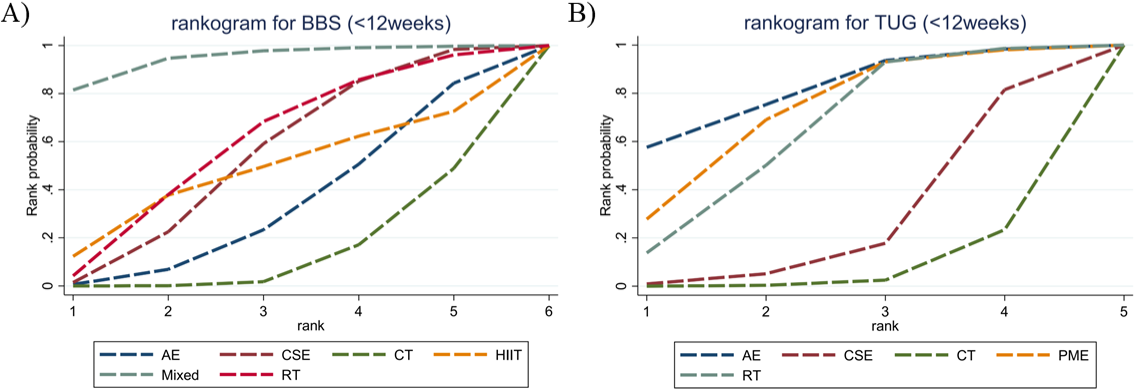


**Figure S6:** SUCRAs for each outcome indicator in the <12-week intervention subgroup. A) BBS; B) TUG.


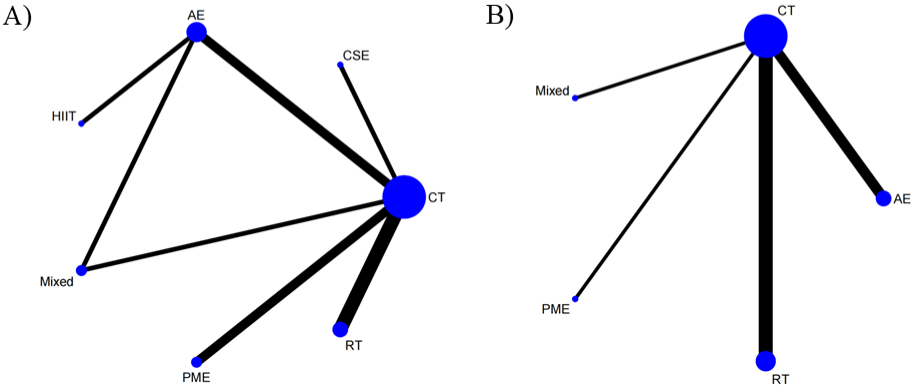


**Figure S7:** Network plot of the effectiveness of each intervention (duration ≥12 weeks) on BBS, TUG, and MoCA Scores. A) BBS; B) TUG. The points in the figure represent various interventions, with the size of each point indicating the sample size. The lines connecting two points illustrate direct comparisons between different interventions, where a thicker line signifies a greater number of corresponding studies.


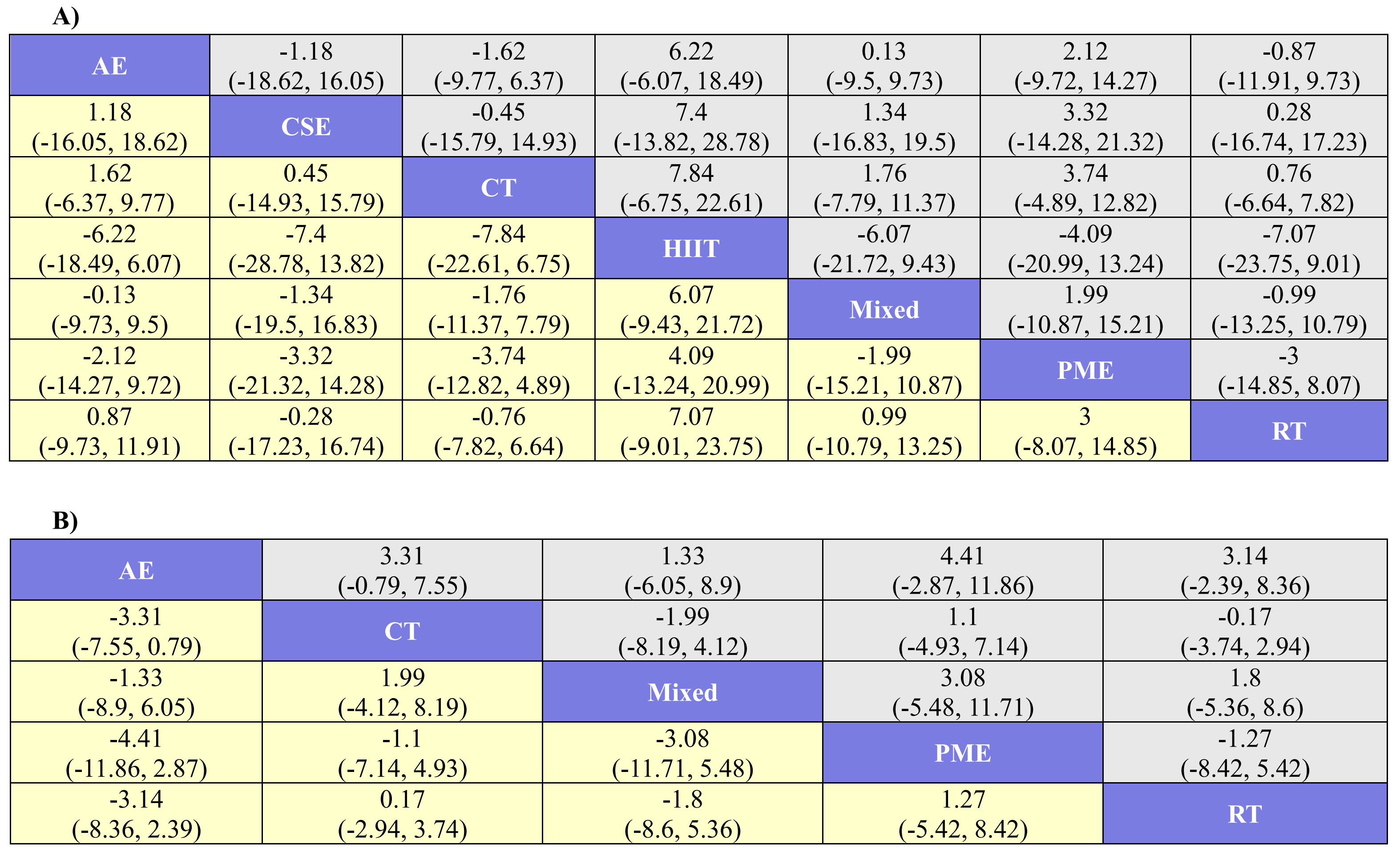


**Figure S8:** League tables for each outcome indicator in the ≥12-week intervention subgroup A) BBS; B) TUG.


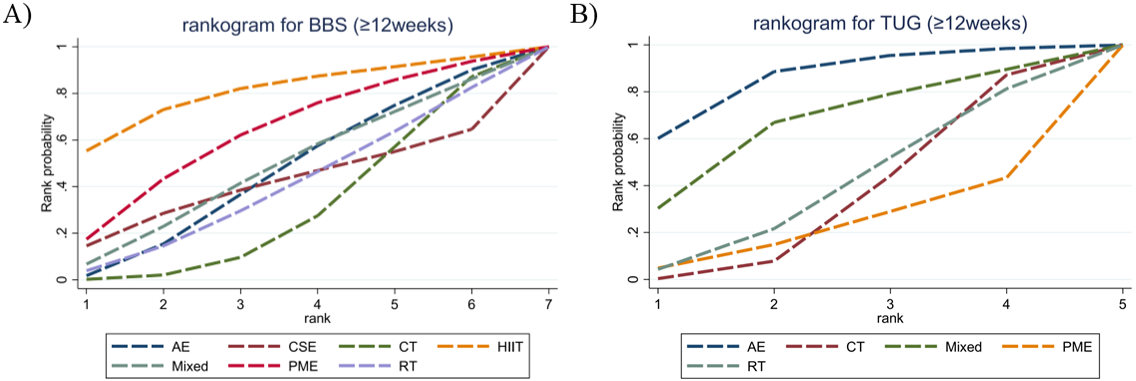


**Figure S9:** SUCRAs for each outcome indicator in the ≥12-week intervention subgroup. A) BBS; B) TUG.
